# Insult-induced adaptive plasticity of the auditory system

**DOI:** 10.3389/fnins.2014.00110

**Published:** 2014-05-23

**Authors:** Joshua R. Gold, Victoria M. Bajo

**Affiliations:** Department of Physiology, Anatomy and Genetics, University of OxfordOxford, UK

**Keywords:** cochlea, auditory cortex, hearing loss, neural plasticity, tinnitus, peripheral insult

## Abstract

The brain displays a remarkable capacity for both widespread and region-specific modifications in response to environmental challenges, with adaptive processes bringing about the reweighing of connections in neural networks putatively required for optimizing performance and behavior. As an avenue for investigation, studies centered around changes in the mammalian auditory system, extending from the brainstem to the cortex, have revealed a plethora of mechanisms that operate in the context of sensory disruption after insult, be it lesion-, noise trauma, drug-, or age-related. Of particular interest in recent work are those aspects of auditory processing which, after sensory disruption, change at multiple—if not all—levels of the auditory hierarchy. These include changes in excitatory, inhibitory and neuromodulatory networks, consistent with theories of homeostatic plasticity; functional alterations in gene expression and in protein levels; as well as broader network processing effects with cognitive and behavioral implications. Nevertheless, there abounds substantial debate regarding which of these processes may only be sequelae of the original insult, and which may, in fact, be maladaptively compelling further degradation of the organism's competence to cope with its disrupted sensory context. In this review, we aim to examine how the mammalian auditory system responds in the wake of particular insults, and to disambiguate how the changes that develop might underlie a correlated class of phantom disorders, including tinnitus and hyperacusis, which putatively are brought about through maladaptive neuroplastic disruptions to auditory networks governing the spatial and temporal processing of acoustic sensory information.

The adult mammalian auditory system shows a remarkable degree of plasticity in a variety of contexts, and at a number of processing levels, manifesting as changes in the central representation of acoustic stimuli. These modulatory processes in the adult auditory brain are thought to be crucial to the performance and learning of ecologically relevant behaviors (Froemke and Martins, 2011; King et al., 2011), as well as being differentially affected during active and passive listening (Pienkowski and Eggermont, 2011). The types of changes observed in these contexts tend to be adaptive if behavior is assessed. However, as previously reviewed (Salvi et al., 2000; Syka, 2002), the mechanisms that develop following a sensorineural auditory insult may represent a unique assemblage, specific to abnormal or damaged sensory input. For example, cholinergic modulation of auditory cortical plasticity plays a key role in driving tonotopic reorganization (Kilgard and Merzenich, 1998) or training-related plasticity (Reed et al., 2011), including regaining acoustic spatial localization after unilateral conductive hearing loss (Leach et al., 2013). However, the cholinergic system is seemingly extraneous to cortical tonotopic plasticity following permanent cochlear damage (Kamke et al., 2005). Therefore, the various outcomes of auditory insults that affect normal cochlear function may constitute an important route for investigating the capacity of the mammalian brain for dealing with disrupted sensory inputs.

The burden posed by hearing-related trauma represents a significant challenge to healthcare services globally, with the rate of hearing impairment approaching ~40% in some adult populations (Agrawal et al., 2008; Nondahl et al., 2011), while the risk of hearing impairment appears to be climbing amongst younger cohorts (Niskar et al., 2001). Each of these epidemiological observations suggest that hearing loss and auditory trauma-related symptoms are liable to affect larger proportions of the future population. There is thus impetus motivating the investigation of the underlying mechanisms that might be responsible for peripheral and the central changes that cause abnormalities in spectrotemporal processing, which in day-to-day life can have a substantial impact on speech perception and auditory scene analysis.

Furthermore, a link between auditory disruption and phantom percept generation has been appreciated for years (e.g., Axelsson and Sandh, 1985). More recently, interest in the possible central origins of such phantoms has been sparked by suggestions that maladaptive neuroplasticity may be responsible for these percepts (Eggermont and Roberts, 2004), even in the absence of any audiometrically identifiable hearing loss (Schaette and McAlpine, 2011). Clearly, the instantiation of tinnitus represents a problem: both for its human sufferers, substantially decreasing quality of life in some patients, particularly given the dearth of effective, broadly applicable treatments available (Baguley et al., 2013; Langguth et al., 2013); but also for researchers investigating the neurobiological mechanisms of the disease, particularly given the little consensus regarding the factors that induce and then maintain the disease chronically (Kaltenbach, 2011; Knipper et al., 2013; Noreña and Farley 2013; Roberts et al., 2013).

To this end, the current review will seek to examine the confluence of factors related to auditory insults from a bottom-up perspective, addressing changes in peripheral function associated with mechanically-, acoustically-, pharmacologically-, and aging-mediated disturbances. The central consequences of these changes to hearing receptor function will then be evaluated: at the level of the single neuron, its local circuitry, as well as the global systemic effects likely to be responsible for behavioral changes that have been categorized in animal models of auditory trauma (Table 1). By occupying a non-hypothesis-driven position in considering the available data across a variety of fields, certain important functional commonalities and/or exceptions will hopefully come to light.

**Table 1:** **Summary of the changes observed at the various levels of the auditory pathway after different types of auditory insults**.

**Category of insult**	**Details of insult**	**Changes in auditory periphery**	**Level of auditory system**	**Cellular expression changes**	**Neuronal activity changes**	**Receptive field changes**	**Behavioral changes**
Acoustic trauma	Tones	↑ Threshold^1^	Ventral cochlear nucleus	↑ GABRA1, GRIN1, RAB3GAP1, KCNK15^6^	↑ SFR (high-CF units)^7^	↓ Inhibitory receptive field, ↑ excitatory activity^8^	
		↓ Hair cell integrity^2^^,^^3^					
		↓ Ribbon synapse density^4^^,^^5^					
			Dorsal cochlear nucleus		↑ SFR^9^^,^^10^	Tonotopic remapping toward lower edge frequencies^14^	
					↑ Bursting activity^9^^,^^11^		
					↑ Response threshold^10^^,^^12^		
					↑ Driven activity^13^		
			Brainstem/midbrain		↓ ABR amplitude recovery^4^^,^^5^		↓ Operant detection performance^4^^,^^5^
					↑ ABR evoked amplitude^15^		↓ GPIAS efficacy^15^
			Inferior Colliculus	↑ BDNF^16^	↑ SFR^18^:	Tonotopic remapping toward lower edge frequencies^23^	
				↓ GAD65 (acute)^17^	2–6 weeks: subject to ANF lesion^19^;	↑ Gap detection thresholds	
				↑ Muscimol binding^17^	8–12 weeks: stable^20^;		
				↓ GABRA1, KCNK15; GLRA1, GRIA2, GRIN1, RAB3GAP1 (acute)^6^	diminished by DCN removal^21^		
					↑ RLF gain^22^		
			Medial geniculate body				
			Auditory cortex	↓ GAD67^24^	N.C./↓ evoked rate^15^	Tonotopic remapping^15^^,^^24^^,^^26^^,^^29^^–^^31^	↓ GPIAS efficacy^15^
				↓ Arg3.1/Arc^4^^,^^5^,^16^	↓ synaptic excitation, inhibition (low-CF)^24^	↓ Gap detection thresholds^32^	↓ Conditioned place preference bias^24^
				↓ BDNF^16^	↓ synaptic inhibition^24^		
				Modified dendrite and spine morphology^25^	↑ RLF monotonicity^26^		
				↑ Membrane excitability^25^	↑ SFR^27^^,^^28^		
					↑ Synchrony^27^^,^^28^		
	Noise	↑ Threshold^33^^,^^34^	Ventral cochlear nucleus	↓ Presynaptic fiber density^41^	↑ RLF slope^44^		
		↓ Hair cell integrity^35^		N.C. c-Fos labeling^42^			
		↓ Ribbon synapse density^36^		↑ GAP-43 expression (presynaptic)^43^			
		↓SGC density (long term)^37^^,^^38^					
		↓RLF gain^39^^,^^40^					
			Dorsal cochlear nucleus	↑ c-Fos^42^	↑ SFR^47^^,^^48^	↑ Bimodal facilitation^48^	↓GPIAS efficacy^48^^,^^50^,^51^
				↑/↓ GlyR mRNA + protein^45^	↑ Driven activity^48^	↑Anti-Hebbian STDP^50^	
				Fiber degeneration^46^	↑ Response threshold^49^		
			Brainstem/midbrain	↑Transneuronal degeneration^46^	↑ Wave 5 amplitude^36^		↑ ASR amplitude^36^
					↑ ABR amplitude^51^		↓ GPIAS efficacy (near-gap)^36^^,^^51^
					↑ ABR latency^51^		
			Inferior Colliculus	N.C. c-Fos^52^	↑/↓ RLF gain^53^		
				↑ c-Fos^42^			
				↑ Transneuronal degeneration^46^			
			Medial geniculate body	N.C. c-Fos^42^			
			Auditory cortex	↑ c-Fos^42^	↑/↓ Evoked amplitudes^54^	Tonotopic remapping^56^	↓ GPIAS efficacy^56^
				N.C. Arg3.1/Arc (100 dB exposure)^52^	↑ SFR^55^^,^^56^	↓ Surround inhibition without tonotopic remapping^57^	
					↑ Synchrony^55^^,^^56^		
					↑ Evoked spike rate^56^		
Mechanical trauma	Complete	Total deafferentation	Ventral cochlear nucleus	↑ Fiber degeneration^58^^,^^59^	Loss of SFR^70^^,^^71^		
				↑ GAP-43^41^			
				Complex changes in glutamate^60–63^, GABA^64^, and glycine^64–67^ pharmacology			
				↑ ERK^68^; complex changes in BDNF & NT-3^69^, and c-JUN & SAPK^68^			
			Dorsal cochlear nucleus	↑ Fiber degeneration^58^^,^^59^	No loss of SFR^70^		
				↑ GAP-43^41^			
				Complex changes in glutamate^60–63^, GABA^64^, and glycine^64–67^ pharmacology			
				↑ ERK^68^; complex changes in BDNF & NT-3^69^, and c-JUN & SAPK^68^			
			Brainstem/midbrain	↑ Transneuronal fiber degeneration^58^			
				Complex changes in glutamate^60–63^, GABA^64^, and glycine^64–67^ pharmacology			
				↑ ERK^68^; complex changes in BDNF & NT-3^69^, and c-JUN & SAPK^68^			
			Inferior colliculus	↑ Transneuronal fiber degeneration^58^		↑ Contralesional sensitivity^73^	
				↓ GAD67^72^, ↓ GlyRα1^72^			
			Medial geniculate body		↓Thalamocortical synchrony^74^		
			Auditory cortex	Complex changes in immediate early-, plasticity related-, and neurotransmission related-gene expression^75^		N.C. Gap detection thresholds to contralesional acoustic stimulation^76^	
	Partial	Organ of corti and AN can be preserved^77^^,^^78^	Ventral cochlear nucleus	↓ GABRA1, GAD1, KCNK15^79^	↑ SFR^79^	↑ Contralesional sensitivity^71^	
			Dorsal cochlear nucleus	↑ Fiber degeneration^58^	↑ Response thresholds/loss of responsiveness^14^	Tonotopic remapping^14^	
			Brainstem/midbrain				
			Inferior colliculus	↓ GABRA1^80^	↑ Response thresholds/loss of sharp tuning^78^^,^^81^^–^^83^	↑ Contralesional sensitivity^81^	
						Tonotopic remapping^78^^,^^81^^–^^83^	
			Medial geniculate body		N.C. Response thresholds^84^		
			Auditory cortex		N.C. Inhibitory gain^85^	Tonotopic remapping^86–88^, not reliant on BFB cholinergic function^88^	
					N.C. Response thresholds^86^^,^^87^		
Drug trauma	Ototoxicity	Extensive OHC loss, often with basal IHC degeneration^89–93^	Ventral cochlear nucleus	↓ Glutamate/aspartate (CC)^101^			N.C. Behavioral audiogram with moderate IHC loss (CC)^104^
		Ribbon synapse disruption^94^		↑ GAP-43^98^			
				↓ VGLUT1^102^			
				↓ Glycine-+’ve puncta^103^			
		Loss of ANF sharp tuning^95^	Dorsal cochlear nucleus	↓ VGLUT1^102^^,^^105^	↑ SFR^92^^,^^106^		
				↑ VGLUT2^102^^,^^105^			
				↓ Glycine-+’ve puncta^103^			
		Carboplatin in the chinchilla: selective IHC ablation^96^^,^^97^, with OHC loss at high dose^98^; reduction in compound action potential gain^99^^,^^100^	Brainstem/midbrain	↓ Glycine-+’ve puncta^107^			
			Inferior colliculus		↑ SFR^108^	↑ Contralesional sensitivity (unilateral cochlear injection)^111^	↑ Conditioned reflex at 1 kHz^108^
					↑ Synchrony^108^		
					↓ Synchrony (CC)^108^		
					N.C. RLF gain or amplitude (CC)^109^		
					↓ RLF amplitude (CC)^99^^,^^100^		
					↓ RLF monotonicity (CC)^110^		
			Medial geniculate body				
			Auditory cortex		↑ RLF gain (CC)^99^	Tonotopic remapping^112^^,^^113^	
						↑ Contralesional sensitivity (unilateral cochlear injection)^111^	
						↑ Crossmodal sensitivity^114^^,^^115^	
	Salicylate	↑ ANF SFR^116^^,^^117^	Ventral cochlear nucleus				
		↑ CAP threshold^118^					
		↓ CAP gain^119^^,^^120^					
		↓ DPOAE gain^118^^,^^119^					
			Dorsal cochlear nucleus				
			Brainstem/midbrain				
			Inferior colliculus	↑ GAD^121^	↑ Response threshold^122^		
				↑ Muscimol binding affinity^121^	↑ SFR^123^		
				↓ Muscimol binding sites^121^	↓ RLF gain (RW application)^120^		
			Medial geniculate body				
			Auditory cortex		↑/↓ SFR (systemic)^124–128^	Tonotopic remapping toward 10–20 kHz^119^	↓ GPIAS efficacy^120^^,^^129^ (50 dB)^122^
					↓ SFR (brain application)^124^	↑ Gap detection threshold^122^	↑ ASR amplitude^129^
					↑ RLF gain^120^^,^^127^		
					↓ RLF gain^120^		
Aging		↓ IHC-ANF ribbon synapse density^130^^,^^131^	Ventral cochlear nucleus	↓ Glycine^147^^,^^148^	↑ Response threshold^150^		
		↓ Spiral ligament & stria vascularis integrity^132–136^		↓ Strychnine binding sites^148^^,^^149^			
		↓ SGC density^37^^,^^38^^,^^130^^,^^132^^,^^136^^–^^142^					
		↓Hair cell integrity^130^^,^^132^^,^^139^^,^^142^^–^^146^					
			Dorsal cochlear nucleus	↓ Glycine^147^^,^^148^	↑ Response threshold^152^^,^^153^		↓ GPIAS efficacy^151^
				↓ Strychnine binding sites^148^^,^^149^,^151^	↑ RLF monotonicity & gain^152^^,^^153^		
			Brainstem/midbrain		↑ ABR latency^154^		
					↓ Brainstem modulation coding in noise^155–157^		
			Inferior colliculus	↓ Glycine^147^	↓ Efficacy of fast AM encoding^161–163^		
				↓ GABA^158^^,^^159^	↓ Gap encoding efficacy ^164^		
				↓ GAD65/67^160^			
			Medial geniculate body	↓ Glutamate^158^	↓ sIPSC (MGBv), ↑sIPSC(MGBd)^165^		
				↓ GABA^158^			
				↓ Glycine^147^			
				↓GAD67^165^			
				↓ Extracellular GABA_A_R^165^			
			Auditory cortex	↓ Parvalbumin staining^166^^,^^167^	↑ SFR^171^	↑ Gap detection threshold^173^	↓ Temporal processing task performance^166^
				↓ GAD65/67^160^^,^^168^	↓ Onset latency^172^	↓ Frequency receptive field encoding efficacy^174^	
				Modified GABA_A_R subunit composition^169^^,^^170^		↓ Spatial receptive field encoding efficacy^171^^,^^172^	

*N.C., No change (compared with control); SFR, spontaneous firing rate; GPIAS, gap-mediated prepulse inhibition of the acoustic startle reflex; ABR, auditory brainstem response; CF, characteristic frequency; BFB, basal forebrain; CC, carboplatin treatment in the chinchilla; ANF, auditory nerve fiber; RW, round window; RLF, rate level function; sIPSC, spontaneous inhibitory postsynaptic potential*.

1 Cody and Johnstone (1980),

2 Puel et al. (1998),

3 Kiang et al. (1975),

4 Singer et al. (2013),

5 Rüttiger et al. (2013),

6 Dong et al. (2010a),

7 Vogler et al. (2011),

8 Boettcher and Salvi (1993),

9 Finlayson and Kaltenbach (2009),

10 Kaltenbach and McCaslin (1996),

11 Chang et al. (2002),

12 Zhang and Kaltenbach (1998),

13 Brozoski et al. (2002),

14 Rajan and Irvine (1998),

15 Ahlf et al. (2012),

16 Tan et al. (2007),

17 Milbrandt et al. (2000),

18 Manzoor et al. (2013),

19 Mulders and Robertson (2009),

20 Mulders and Robertson (2011),

21 Manzoor et al. (2012),

22 Salvi et al. (1990),

23 Izquierdo et al. (2008),

24 Yang et al. (2011),

25 Yang et al. (2012),

26 Noreña and Eggermont (2003),

27 Seki and Eggermont (2003),

28 Noreña and Eggermont (2003),

29 Cheung et al. (2009),

30 Seki and Eggermont (2002),

31 Calford et al. (1993),

32 Yin et al. (2008),

33 Hawkins et al. (1976),

34 Liberman and Beil (1979),

35 Spongr et al. (1992),

36 Hickox and Liberman (2014),

37 Lin et al. (2011),

38 Kujawa and Liberman (2009),

39 Heinz et al. (2005),

40 Heinz and Young (2004),

41 Illing et al. (2005),

42 Wallhäusser-Franke et al. (2003),

43 Michler and Illing (2002),

44 Cai et al. (2009),

45 Wang et al. (2009a),

46 Morest and Bohne (1983),

47 Shore et al. (2008),

48 Dehmel et al. (2012b),

49 Ma and Young (2006),

50 Koehler and Shore (2013a),

51 Dehmel et al. (2012a),

52 Mahlke and Wallhäusser-Franke (2004),

53 Willott and Lu (1982),

54 Noreña et al. (2010),

55 Noreña and Eggermont (2006),

56 Engineer et al. (2011),

57 Rajan (1998),

58 Morest et al. (1997),

59 Gentschev and Sotelo (1973),

60 Fyk-Kolodziej et al. (2011),

61 Förster and Illing (1998),

62 Potashner et al. (1997),

63 Godfrey et al. (2014),

64 Suneja et al. (1998b),

65 Potashner et al. (2000),

66 Suneja et al. (1998a),

67 Hildebrandt et al. (2011),

68 Suneja and Potashner (2003),

69 Suneja et al. (2005),

70 Koerber et al. (1966),

71 Bledsoe et al. (2009),

72 Argence et al. (2006),

73 McAlpine et al. (1997),

74 Sun et al. (2009b),

75 Oh et al. (2007),

76 Kirby and Middlebrooks (2010),

77 Nordeen et al. (1983),

78 Snyder et al. (2008),

79 Dong et al. (2009),

80 Dong et al. (2010b),

81 Snyder and Sinex (2002),

82 Snyder et al. (2000),

83 Irvine et al. (2003),

84 Kamke et al. (2003),

85 Rajan (2001),

86 Robertson and Irvine (1989),

87 Rajan et al. (1993),

88 Kamke et al. (2005),

89 Ryan et al. (1979),

90 Nienhuys and Clark (1978),

91 Ryan and Dallos (1975),

92 Kaltenbach et al. (2002),

93 Forge (1985),

94 Liu et al. (2013),

95 Dallos and Harris (1978),

96 Takeno et al. (1994),

97 Wake et al. (1993),

98 Kraus et al. (2009),

99 Qiu et al. (2000),

100 El-Badry and McFadden (2007),

101 Godfrey et al. (2005),

102 Zeng et al. (2009),

103 Asako et al. (2005),

104 Lobarinas et al. (2013b),

105 Zeng et al. (2012),

106 Rachel et al. (2002),

107 Buras et al. (2006),

108 Bauer et al. (2008),

109 McFadden et al. (1998),

110 Alkhatib et al. (2006),

111 Popelár et al. (1994),

112 Kakigi et al. (2000),

113 Schwaber et al. (1993),

114 Meredith et al. (2012),

115 Allman et al. (2009),

116 Cazals et al. (1998),

117 Evans and Borerwe (1982),

118 Ruel et al. (2008),

119 Stolzberg et al. (2011),

120 Sun et al. (2009a),

121 Bauer et al. (2000),

122 Deng et al. (2010),

123 Jastreboff and Sasaki (1986),

124 Lu et al. (2011),

125 Ochi and Eggermont (1996),

126 Eggermont and Kenmochi (1998),

127 Yang et al. (2007),

128 Zhang et al. (2011),

129 Sun et al. (2014),

130 Sergeyenko et al. (2013),

131 Stamataki et al. (2006),

132 Hequembourg and Liberman (2001),

133 Buckiova et al. (2007),

134 Ichimiya et al. (2000),

135 Di Girolamo et al. (2001),

136 Kujawa and Liberman (2006),

137 Cohen et al. (1990),

138 Dazert et al. (1996),

139 Engle et al. (2013),

140 Keithley and Feldman (1979),

141 Schmiedt et al. (1996),

142 White et al. (2000),

143 Bielefeld et al. (2008),

144 Chen et al. (2009),

145 Keithley and Feldman (1982),

146 Spongr et al. (1997),

147 Banay-Schwartz et al. (1989a),

148 Willott et al. (1997),

149 Milbrandt and Caspary (1995),

150 Willott et al. (1991),

151 Wang et al. (2009b),

152 Caspary et al. (2006),

153 Caspary et al. (2005),

154 Nozawa et al. (1996),

156 Parthasarathy et al. (2010),

157 Parthasarathy and Bartlett (2011),

158 Banay-Schwartz et al. (1989b),

159 Caspary et al. (1990),

160 Burianova et al. (2009),

161 Shaddock Palombi et al. (2001),

162 Simon et al. (2004),

163 Walton et al. (2002),

164 Walton et al. (1998),

165 Richardson et al. (2013),

166 De Villers-Sidani et al. (2010),

167 Martin del Campo et al. (2012),

168 Ling et al. (2005),

169 Caspary et al. (2013),

170 Schmidt et al. (2010),

171 Juarez-Salinas et al. (2010),

172 Engle and Recanzone (2012),

173 Recanzone et al. (2011),

174 *Turner et al. (2005)*.

## Changes in cochlear function following peripheral insult

Studies of hearing loss predominantly focus on damage inflicted upon the organ of Corti, with the outcomes of direct mechanical (e.g., Robertson and Irvine, 1989; Rajan et al., 1993) or thermal (e.g., Snyder et al., 2008) lesions of the cochlea and its afferents clear in terms of the extent of damage induced. The time scale is also variable, depending upon the idiosyncrasies of other deafening protocols, leading to temporary- (e.g., Calford et al., 1993; Syka and Rybalko, 2000; Dehmel et al., 2012b; but see Kujawa and Liberman, 2009), or permanent threshold shifts, or indeed related to natural aging. Nonetheless, different experimental approaches appear to share certain commonalities that may be responsible for the induction of stereotypical responses in central processing stations.

### The anatomy of cochlear injury

Following an acutely induced acoustic overexposure, the degree of hair cell loss generally scales with the amplitude and duration of the insult. This usually manifests as extensive outer hair cell (OHC) death, with frequency-delimited loss of inner hair cells (IHCs) scaling with the trauma severity (Spongr et al., 1992). Lesion patterns following similar exposure protocols are variable, however. Descriptions have been made of equivalent outer and inner hair cell degeneration (Dolan et al., 1975; Kiang et al., 1975), as well as consistent damage to basally-located OHCs and IHCs regardless of the spectral content or duration of the acoustic trauma (Hawkins et al., 1976; Mulders et al., 2011).

By comparison, disruption of hair cell stereocilia has been observed to occur without inducing apoptosis of the affected hair cells (Liberman and Beil, 1979). This heterogeneous susceptibility to trauma appears to be conserved in certain models of ototoxicity, such as aminoglycoside antibiotic exposure (Forge, 1985), or that induced by platinum-derived cancer treatment drugs (specifically cisplatin and carboplatin) (Yorgason et al., 2006). Certainly, these treatments are recognized for their potentially devastating side-effects in human patients. Interestingly, while each of these latter, platinum-based agents leads primarily to OHC destruction, a lack of dose dependency has been reported, often leading to variable degrees of OHC damage for a range of exposure concentrations (Kaltenbach et al., 2002). Notable is the unique case of the chinchilla, which suffers a severe, and often, complete, ablation of the IHC population by carboplatin (Wake et al., 1993; Takeno et al., 1994), affecting OHCs only when doses verge on systemic toxicity, at which point total hair cell losses are substantial (Kraus et al., 2009).

It is constructive to highlight an equivalency between losses developed following acute trauma, and those acquired during aging. Age-related changes affect the cochlea in a stereotyped, multifactorial manner (Schuknecht, 1964; Schuknecht and Gacek, 1993; Ohlemiller, 2004), with hearing deterioration being characterized according to the underlying pathology of the receptor organ. According to Schuknecht's revised categories of the pathology, specific cochlear components may undergo degradation. The types of presbyacusis typically referred to include sensory presbyacusis—a loss of hair cell or organ of Corti integrity; metabolic presbyacusis, indicative of aberrant strial physiology; neural presbyacusis, involving atrophy and/or apoptosis of the afferent spiral ganglion cell fibers in the cochlea; or some combination thereof.

In models of presbyacusis, the relative distribution of hair cell damage appears to vary between species and is often defined by greater OHC impairment (Keithley and Feldman, 1982; Spongr et al., 1997). During the onset of age-related peripheral pathology, progressive deterioration of a number of cochlear structures has also been described: in the stria vascularis (Di Girolamo et al., 2001; Hequembourg and Liberman, 2001) and in the spiral ligament (Ichimiya et al., 2000), as well as a metabolic deterioration of the organ of Corti, which may exacerbate the functional consequences of OHC prestin reduction (Buckiova et al., 2007; Bielefeld et al., 2008; Chen et al., 2009). Ultimately, each of these various cochlear pathologies seems to converge upon auditory neuropathy (Keithley and Feldman, 1979; Cohen et al., 1990; Dazert et al., 1996; White et al., 2000; Engle et al., 2013). This putatively common endpoint, involving complete deafferentation, is likely preceded by functional disturbance of the synaptic interface between spiral ganglion afferents and IHCs (Stamataki et al., 2006; Sergeyenko et al., 2013).

A similar template of neuropathy—in particular concerning damage at the IHC-ANF interface—has been observed in acute experimentally-induced hearing loss, with regions of IHC loss correlated with reactive swelling and peripheral deafferentation of an equivalent spiral ganglion cell (SGC) population (Feng et al., 2012). Akin to the description above, degeneration of SGC perikarya occurs over the weeks to months following trauma, as does selective stria vascularis pathology (Wang et al., 2002). Similarly, inspection of drug ototoxicity has unveiled the development of afferent fiber terminal vacuolization in advance of any discernible IHC damage (Wang et al., 2003). Systemic toxicity is probably responsible for certain aspects of peripheral trauma, although the retraction of peripheral afferents following trauma-mediated terminal swelling has been postulated to be associated with disruption of the IHC ribbon synapse, a specialized glutamatergic structure necessary for synchronized auditory nerve activity (Buran et al., 2010). Importantly, under traumatic conditions, the ribbon synapse structure is capable of mediating an excitotoxic injury of afferent dendrites (Puel et al., 1998; Pujol and Puel, 1999).

**Figure 1:**
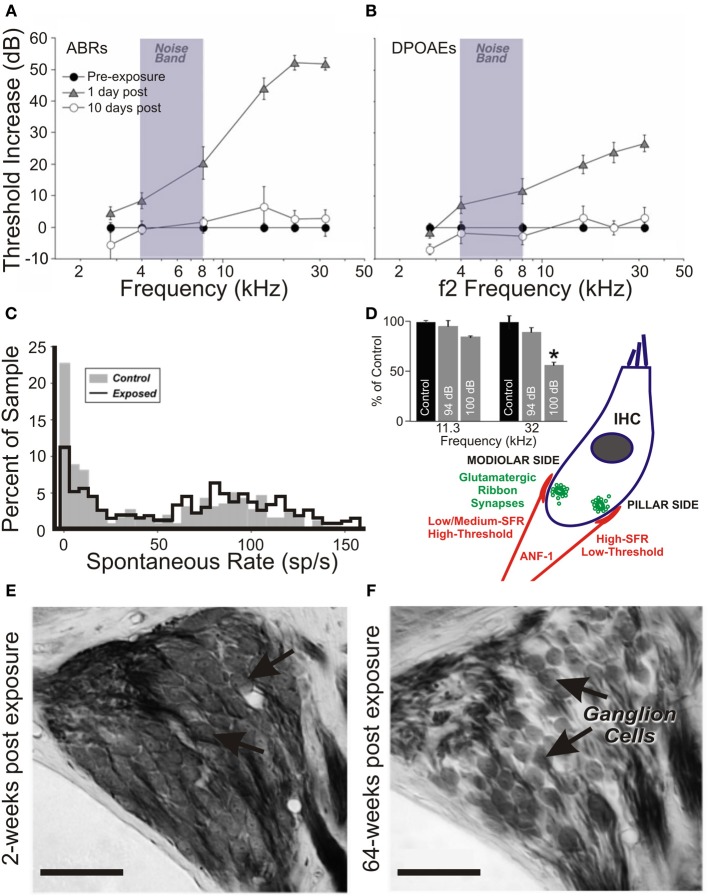
**Insult-induced changes to auditory nerve physiology can occur in the absence of audiometric hearing loss**. Peripheral thresholds measured using auditory brainstem responses **(A)** and distortion product otoacoustic emissions **(B)** following exposure to moderate narrowband noise trauma. Both are elevated in the short term following overexposure (24 h; triangles), however by >7 days thresholds have returned to normal (open circles). Modified with permission from Lin et al. (2011). **(C)** There is a significant shift in the normally bimodal distribution of spontaneous rate in the auditory nerve of exposed animals over a similar time course, leading to underrepresentation of type-1 auditory nerve fibers, coding for frequencies at and above the trauma high-pass corner, with spontaneous rates <20 spikes/s. Modified with permission from Furman et al. (2013). **(D)** Anatomically, such fibers synapse on the modiolar side of inner hair cells, and typically have a wide dynamic range of response, allowing effective rate coding up to high stimulus intensities. In a mouse model of exposure (inset), these high-threshold type-1 ANF responses are selectively reduced in the acoustic trauma region in the short term following traumatic exposure (8–16 kHz octave-band noise, 100 dB SPL), but not for exposure to a non-traumatic stimulus (94 dB SPL). Modified with permission from Hickox and Liberman (2014). **(E,F)** Histological comparison between the spiral ganglia basal turns of mice in the short- **(E)** and long- **(F)** terms following trauma finds that this pattern of high-threshold response loss precipitates ongoing SGC apoptosis. Scale bars 50 μm. Modified with permission from Kujawa and Liberman (2009). ^*^p ≤ 0.01.

Ribbon synaptopathy has been noted in the absence of overt cellular morphological disruption (Liu et al., 2013), with recent evidence indicating that it may well be a predictable consequence of cochlear trauma (Rüttiger et al., 2013; Singer et al., 2013). Observations of ribbon synaptopathy following even mild acoustic exposure (Maison et al., 2013) are particularly compromising from a functional perspective, since cochlear degeneration may have unwanted consequences at much later time points after the initial insult (Kujawa and Liberman, 2006, 2009; Sergeyenko et al., 2013) (Figure 1). How, then, are the various structural consequences of cochlear trauma to be reconciled with functional changes occurring in auditory nerve afferent signaling?

### The assessment and outcomes of peripheral trauma

Audiogram-based assessment is often capable of detecting hearing loss defined in terms of OHC dysfunction and/or threshold elevation, and is used extensively to define the consequences of cochlear trauma as “permanent” or “temporary” threshold shifts. Typically, this is via inspection of waveforms of the auditory brainstem response (ABR) or auditory nerve fiber compound action potential (CAP), in conjunction with distortion product otoacoustic emissions (DPOAEs), or simply via self-reporting in human patients. By contrast with the threshold losses mediated by mixed OHC/IHC lesions (Cody and Johnstone, 1980; Liberman and Dodds, 1984a) or OHC loss alone (Dallos and Harris, 1978), the pattern of extensive cochlear damage induced by carboplatin administration in the chinchilla manifests as a time-dependent reduction in the CAP amplitude, often without pathological modulation of DPOAEs or ABR synchrony (El-Badry and McFadden, 2009), and only minor elevation (~9 dB) of electrophysiological thresholds (El-Badry and McFadden, 2007). This is reflected functionally in the preservation of behavioral audiograms collected from carboplatin-exposed chinchillas (Lobarinas et al., 2013b). It is certainly concerning that IHC pathology can extend to include up to 80% of the cochlear population, without similarly drastic changes in measures provided by commonly-used diagnostic tools.

Similarly, long-term degeneration of SGCs has been noted in aged mice with intact IHCs and permanently raised ABR thresholds (Kujawa and Liberman, 2006). More recently, a type of insidious pathology has been characterized following an apparently limited, acute hearing loss, previously thought to have minimal ongoing ramifications at the periphery (Figure 1). Inspection of mouse cochleae without evidence of raised thresholds (on ABRs or DPOAEs), nor with any overt structural damage to OHC or IHC populations, revealed IHC-specific ribbon synaptopathy within 24 h of acoustic trauma, manifesting as suprathreshold reductions in ABR amplitude at the affected cochleotopic frequencies (Kujawa and Liberman, 2009). More surprisingly, late-stage (>2 years) cochleography exposed a reduction by ≤50% of SGC density, regardless of the apparent health of the organ of Corti. In addition to these findings being replicated in the guinea pig (Lin et al., 2011), afferent degeneration coming about in this “silent” deafness seems only to affect those auditory nerve fibers encoding high-intensity stimuli (Furman et al., 2013).

Diagnostic issues would undoubtedly be associated with detecting this type of selective insult to high-threshold, low-spontaneous-rate fibers, especially since classical studies have correlated hearing loss with reductions in afferent spontaneous and evoked firing rates (Lonsbury-Martin and Meikle, 1978; Liberman and Dodds, 1984b). Recent demonstration that the phase-locking capacity of the auditory nerve fibers is diminished in the presence of low-level noise following acoustic trauma (Henry and Heinz, 2012, 2013) suggests that the functional ramifications of high-threshold fiber pathology are liable to be complex and context-specific. Given the multifaceted peripheral sequelae of cochlear trauma, it is important that comprehensive evaluation of auditory afferent function is performed to fully characterize the extent of cochlear pathology following auditory trauma, both clinically and experimentally (e.g., Ahlf et al., 2012; Dehmel et al., 2012a; Gu et al., 2012; Rüttiger et al., 2013).

Of particular use would be the routine implementation of suprathreshold evoked potential-based measurements of peripheral and brainstem auditory function. The collection of ABR data at multiple intensities for each subject (prior to and following trauma in animal experiments) would allow auditory thresholds to be established (either generally, in response to broadband click-train stimuli; or at greater spectral resolution by using tonal or narrowband-passed noise stimuli). Moreover, possible sites of lesion-mediated plasticity could be ascertained by evaluating differences in waveform amplitudes and latencies (either between the trauma population and matched controls, or in repeat measurements of the same subjects under different treatment conditions). Given that ABRs can be obtained repeatedly, they represent a convenient way of tracking, longitudinally, the immediate and longer term effects of commonly-implemented trauma protocols. The collection of such data would greatly improve our understanding of the impact of trauma on brainstem neural populations. For example, data of this kind have been reported in human tinnitus patients with and without audiometric threshold elevations, and have been integral to our ongoing understanding of that pathology (Attias et al., 1996; Kehrle et al., 2008; Schaette and McAlpine, 2011; Gu et al., 2012) (see below).

## Neurochemical and structural changes induced by auditory damage

Induced damage to the peripheral hearing organ causes physiological disruption of neurons at various levels along the auditory neuraxis, affecting not only neurotransmitter communication mechanisms, but also intracellular signaling pathways and metabolism. Although the precise functional impact is yet to be fully elucidated, given the complexity of the central auditory system, plus the extent and temporal features of the various insults examined experimentally, examining how cellular-level processes are affected may enhance our understanding of the higher-order systemic or perceptual outcomes of auditory trauma.

### Activity-related gene modulation

The expression of certain immediate early genes (IEGs), as a surrogate for neuronal metabolic state, has revealed time-varying modulation patterns in the auditory cortex following bilateral cochlear ablation that indicate the short time course necessary for insult-induced genetic mobilization in auditory neurons (Oh et al., 2007). *c-fos* is an IEG induced by stimulus novelty (e.g., Ehret and Fischer, 1991; Rouiller et al., 1992), thought to link neuronal activity with gene transduction associated with plasticity and learning (Carretta et al., 1999; Tischmeyer and Grimm, 1999). In the auditory cortex, *c-fos* is expressed within 1 h following acoustic trauma (Wallhäusser-Franke et al., 2003), in a diffuse manner indicating an acute, widespread (non-tonotopic) hyperreactivity within the cortex (Mahlke and Wallhäusser-Franke, 2004). Enhancements of *c-fos* expression levels were also detected over similar time frames at key subcortical auditory centers (including IC and DCN) following acoustic trauma, suggesting a feedforward upregulation of neural activity throughout the central auditory system.

As an alternative method of modifying cochlear activity, salicylate administration at high doses has been noted to affect hearing thresholds (Cazals, 2000), simultaneously elevating spontaneous activity (Evans and Borerwe, 1982) and diminishing driven response rates (Sun et al., 2009a; Stolzberg et al., 2011) within the auditory nerve. Appropriately, *c-fos* labeling after systemic salicylate injection yielded limited labeling in the non-lemniscal dorsal cortex and external nucleus of the IC (Wallhäusser-Franke, 1997; Mahlke and Wallhäusser-Franke, 2004), with more consistent, dose-dependent expression found at the primary auditory cortex (A1) and anterior auditory field (AAF) (Wallhäusser-Franke et al., 2003).

More recently, region-specific insult-induced changes in the expression of another IEG, Arg3.1/Arc (activity-regulated gene/activity-regulated cytoskeleton-associated protein), have been investigated. Arg3.1/Arc mobilization putatively occurs through brain-derived neurotrophic factor (BDNF)-mediated activation of the MEK-ERK signaling pathway (Ying et al., 2002). Components of this pathway are upregulated in the auditory brainstem following cochlear ablation (Suneja and Potashner, 2003; Suneja et al., 2005) and acoustic trauma (Tan et al., 2007). On examining the effects of salicylate and acoustic trauma on Arg3.1/Arc expression in the adult gerbil, marked divergence from the pattern of *c-fos* labeling is seen, with Arg3.1/Arc upregulation occurring in cortex only (Mahlke and Wallhäusser-Franke, 2004). Furthermore, dissimilarities between the tonotopic and laminar arrangements of Arg3.1/Arc expression were qualitatively related to the type of insult. Systemic salicylate injection enhanced Arg3.1/Arc expression in the high-frequency domain of A1, but predominantly outside of layer IV, thereby suggesting its effects are less likely to be mediated by substantial thalamic input. By contrast, moderate acoustic trauma (at an intensity sufficient to produce long-term cochlear synaptic and afferent dysfunction) yielded a non-layer-specific Arg3.1/Arc expression pattern in a tonotopically constrained manner, effectively matching the cochlear trauma profile (Mahlke and Wallhäusser-Franke, 2004).

**Figure 2:**
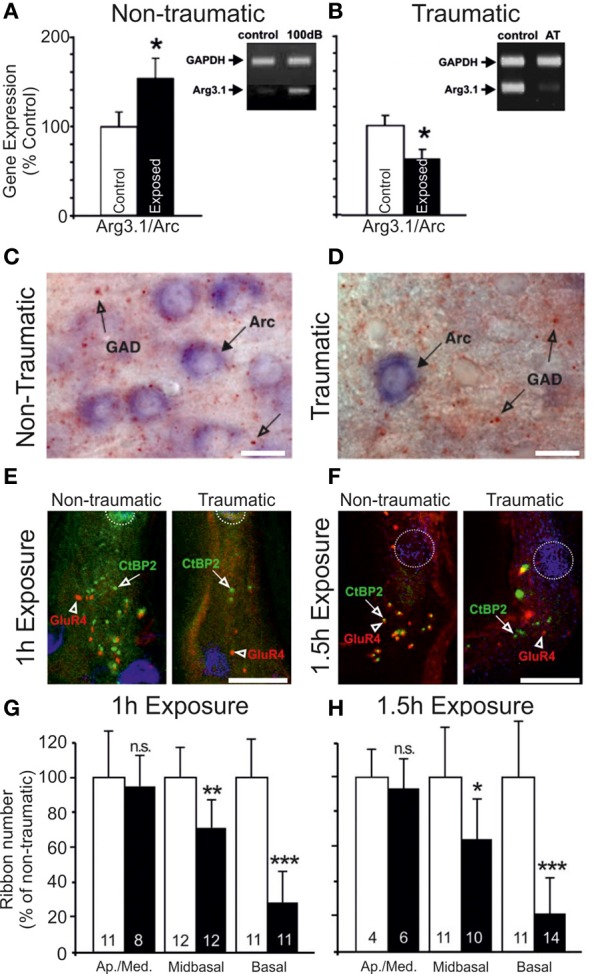
**Changes in the auditory cortex expression of Arg3.1/Arc and cochlear ribbon synapse density related to the degree of acoustic trauma. (A)** Following exposure to acoustic trauma that renders non-permanent threshold elevation (100 dB SPL, 2 h), reverse-transcriptase polymerase chain reaction (RT-PCR) analysis reveals Arg3.1/Arc expression is significantly upregulated in the auditory cortex; **(B)** by contrast, animals overexposed at 115–119 dB SPL for 2 h displayed marked downregulation of Arg3.1/Arc transcipts. Modified with permission from Tan et al. (2007). ^*^p ≤ 0.05. **(C,D)** This relative downregulation is illustrated by immunohistochemical labeling of Arg3.1/Arc mRNA in the auditory cortex of animals exposed to a similar trauma (120 dB SPL, 2 h). Glutamate decarboxylase puncta are also indicated. Modified with permission from Singer et al. (2013). When animals were acoustically overexposed for either **(E)** 1 h or **(F)** 1.5 h, staining in the cochlea for IHC ribbon synapses (CtBP2, green, open arrows) indicated a subset of animals with marked reduction in ribbon synapse density, illustrated here for the mid-basal turn of the cochlea. IHC nuclei are labeled with DAPI (blue, circled); glutamate receptor subunit GluR4 protein labeled (red, open arrowheads). Scale bars 10 μm. **(G,H)** For each exposure protocol, a subset of animals could be categorized according to significant proportional reductions in midbasal and basal cochlear IHC ribbon synapse densities, relative to exposed animals without this pathology. Open bars = controls; filled bars = exposed. Numbers in each bar correspond to respective *n*. Modified with permission from Rüttiger et al. (2013). ^*^p ≤ 0.05; ^**^p ≤ 0.01; ^***^p ≤ 0.001.

In more recent studies of acoustic trauma, it was intriguing that those exposed subjects with demonstrable IHC ribbon synapse degradation displayed Arg3.1/Arc mobilization that appeared to be down, not up (Tan et al., 2007; Rüttiger et al., 2013; Singer et al., 2013) (Figure 2). Indeed, this bidirectional Arg3.1/Arc modulation is inversely correlated with the degree of BDNF and *c-fos* expression in the spiral ganglion, for periods spanning 3 h to 6 days after acoustic exposure (Tan et al., 2007); Arg3.1/Arc was downregulated in the cerebral cortex for at least 14–30 days thereafter (Rüttiger et al., 2013; Singer et al., 2013). Although the functional consequences of long-term up- or downregulation following auditory trauma remain uncertain, Arg3.1/Arc is thought to play a key role in controlling AMPA-type glutamate receptor distribution during homeostatic plasticity (e.g., Shepherd et al., 2006; Bramham et al., 2010; Gao et al., 2010; Béïque et al., 2011).

### Excitatory signaling function

On the basis of those data, it might be expected that significant alterations in glutamatergic function would be observed after the auditory insult—however, characterization of glutamate receptor pharmacology in the deafferented auditory cortex remains conspicuously underrepresented in the literature. As such, the observed modulations in Arg3.1/Arc mobilization (reviewed in Knipper et al., 2010, 2013) might be functionally linked with those changes in cortical neurotransmission and neuronal response properties under similar exposure conditions, or rather might simply correlated with the induction of certain forms of hearing loss, without attendant regulation of homeostatic plasticity mechanisms (Bramham et al., 2010).

Changes in glutamatergic metabolism have been better documented subcortically, such as the small, non-significant reduction of glutamate concentrations in the medial geniculate body (MGB), inferior colliculus (IC) and cochlear nucleus (CN) of a rodent model of presbyacusis (Banay-Schwartz et al., 1989b). Cochlear ablation, by contrast, induces enhanced glutamate metabolism, manifesting as elevated release and uptake, and concomitant with a putative compensatory strengthening of excitatory processing following extensive afferent fiber death (Potashner et al., 1997; Illing et al., 2005), as levels of glutamate and aspartate in the CN correlate with the degree of cochlear damage (Godfrey et al., 2005, 2014). While the proportional expression of glutamate vesicular transporter (VGLUT) subtypes has been reported to undergo adjustment in the insult-exposed CN (Fyk-Kolodziej et al., 2011), work specifically directed at such changes in the dorsal cochlear nucleus (DCN) has unveiled marked crossmodal reweighing. By assigning VGLUT subtypes 1 and 2 to auditory and non-auditory inputs, respectively (Zhou et al., 2007), the upregulated representation of non-auditory afferents in the fusiform layer of the DCN following cochlear trauma was revealed (Zeng et al., 2009, 2012).

Furthermore, in the short term, mRNAs encoding glutamatergic AMPA and NMDA-type receptor subunits are reduced in the central nucleus of the IC (CNIC) contralateral to a partial mechanical lesion of the cochlea (Dong et al., 2009, 2010a). Although these changes are only transient, they may be sufficient to more chronically disrupt cellular excitability elsewhere in the central auditory system. Such an outcome might follow in parallel within the auditory brainstem, given the reported remodeling of AMPA binding and NMDA-receptor subunit expression in the ventral cochlear nucleus (VCN) (Förster and Illing, 1998; Suneja et al., 2000). In spite of these efforts, many details of post-traumatic glutamate pharmacology remain to be elucidated.

### Inhibitory signaling function

A more comprehensive understanding has been gained of inhibitory changes, which, in addition to the reactive changes in glutamate release and signaling subcortically, may precipitate a disruption of the balance of excitation and inhibition. Even at the earliest stages of auditory processing, inhibitory inputs are affected, failing to recover to pre-lesion levels in the ventral CN (Hildebrandt et al., 2011). In line with these disruptions, reductions in the density of glycine-immunoreactive puncta (Asako et al., 2005) and glycine receptor subunit protein (Wang et al., 2009a) have each been reported throughout the cochlear nucleus (CN). Such changes develop alongside altered strychnine binding and glycine metabolism in the ipsilateral CN and superior olivary complex, earlier described in the deafened guinea pig (Suneja et al., 1998a,b) and rat (Buras et al., 2006), suggesting a sustained post-deafening hyperexcitability (Potashner et al., 2000). These inhibitory disruptions occurring in a number of auditory brainstem nuclei are, perhaps, not unexpected, since previous work has demonstrated significant reductions in both the levels of glycine (Banay-Schwartz et al., 1989a; Willott et al., 1997; Wang et al., 2009b) and glycine/strychnine binding sites (Milbrandt and Caspary, 1995; Willott et al., 1997) in rodent models of presbyacusis.

Possibly the most heavily scrutinized locus for such representative disruptions to the auditory pathway is the inferior colliculus (reviewed in Caspary et al., 2008; Ouda and Syka, 2012). Aged rodents display decreases in free GABA concentrations throughout the IC (Banay-Schwartz et al., 1989b), as well as in evoked GABA release and GABAergic cell density (without modification of GABAergic cellular morphology) (Caspary et al., 1990), and reduced labeling for GAD65 and GAD67 (Burianova et al., 2009). GAD67 is also reduced following acute, complete deafferentation (Argence et al., 2006), although when the extent of damage is limited to certain frequency domains, the downregulation of transcripts coding for GABA_A_ receptor subunit 1 and GAD1, respectively, were matched bilaterally by an effective reduction in GABAergic receptor protein expression in a tonotopically limited fashion (Dong et al., 2010b). It is noteworthy that GAD disruptions may occur only transiently (Milbrandt et al., 2000), indicating the possibility for the metabolic recovery of GABAergic signaling if trauma severity is limited. Indeed, enhanced collicular GABA immunoreactivity (Tan et al., 2007) (Figure 2) and GAD65 levels (Bauer et al., 2000) each have been described, possibly compensating for elevated network activity following auditory trauma.

A comparable GABAergic dysregulation is also seen cortically. A reduction in free GABA concentration is found in aged rat cortex (Banay-Schwartz et al., 1989b), and is possibly related to reductions in the levels of GAD65/67 mRNA, and GAD67 protein across cortical layers. Those observations were reported in two different rat strains that, importantly, display divergent peripheral symptoms as a function of aging but shared central inhibitory sequelae (Ling et al., 2005; Burianova et al., 2009). Indeed, lower GABA density throughout the auditory cortex appears to be co-determined by age and hearing loss, since GAD65 expression is also reduced after acoustic trauma in younger adult animals (Yang et al., 2011). Moreover, the receptor subunit composition of GABA_A_ receptors is heterogeneously modified in aged rats (Schmidt et al., 2010), with a 30% reduction in the wild-type α1 subunit compared with young controls, as well as in certain subtypes of β- and γ-subunits (Caspary et al., 2013). In total, then, despite the picture of post-traumatic auditory neurochemistry standing broadly incomplete, certain characteristic changes appear to occur regardless of insult type. The extent to which these affect physiological function and neural response properties will therefore require examination. Additionally, an improved understanding of the neuropharmacological correlates of auditory trauma would be beneficial in the development of patient treatment related to the compensation for those changes (Schreiber et al., 2010; Mukherjea et al., 2011).

## Neuronal spontaneous and evoked response properties undergo plastic modulation during and after auditory insults

It is worthwhile considering that, even in the absence of acoustic stimulation, the net firing rate of the auditory nerve is particularly high in healthy animals (e.g., Kiang et al., 1975; Liberman and Dodds, 1984b); as such, cochlear exposure to various insults affecting peripheral and/or central function (particularly pharmacological and age-related) might be predicted to have profound effects on baseline levels of activity throughout the auditory system. Consequently, it is important to evaluate how single cells in the auditory system, when faced with abnormal synaptic inputs, may dynamically modify their activity and, by association, their properties related to the processing of acoustic stimuli.

### Auditory nerve

The activity of auditory nerve fibers (ANFs) changes in response to various experimental insults. Interpretation of data concerning post-traumatic amplitude transfer functions and temporal coding under diminished hearing conditions has been informed by classical physiological studies. In early experiments, wholesale mechanical cochlear ablation was shown to yield a complete loss of spontaneous activity within the auditory nerve and its afferent terminals within the ventral cochlear nucleus (VCN) (Koerber et al., 1966). This ultimately produced substantial fiber loss and density reduction throughout the VCN (Gentschev and Sotelo, 1973; Morest and Bohne, 1983; Morest et al., 1997). Under such circumstances, it is apparent that some ANFs remain functionally normal, even if the unlikelihood of recording from an active fiber scales with the degree of IHC damage (Wang et al., 1997). In such cases, functional normality is apparently contingent upon modification of the electrical properties of individual axons (Fryatt et al., 2011), and the survival of the OHC population. The latter is particularly necessary in maintaining sharp, low threshold tuning at the fibers' respective characteristic frequencies (CF) (Kiang et al., 1975; Dallos and Harris, 1978).

**Figure 3:**
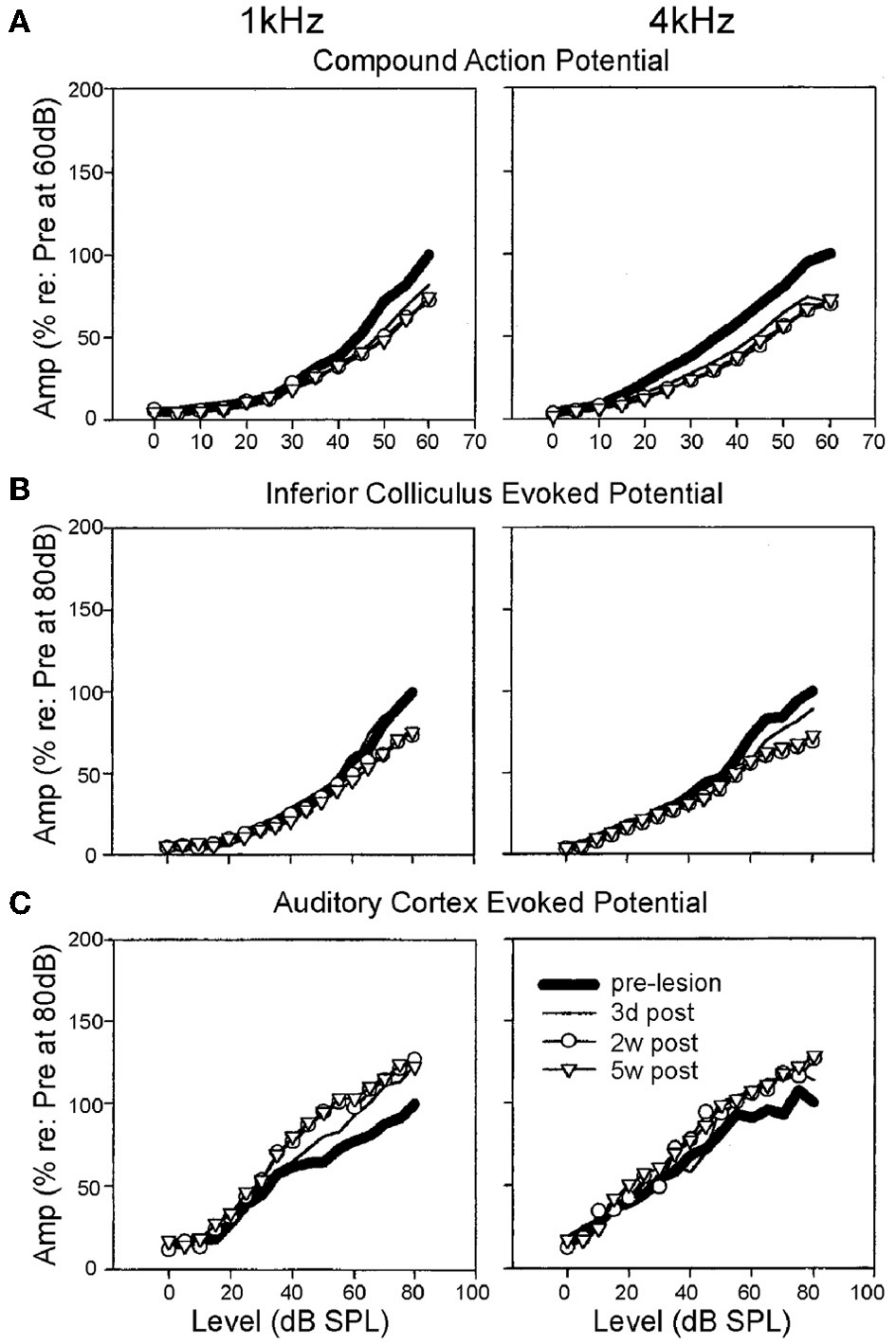
**Specific elevation of auditory evoked rate level functions after selective inner hair cell ablation**. Following systemic injection of carboplatin (various doses) into chinchillas, the selective IHC lesion that develops was evaluated by chronic electrode implantation with measurement of evoked field response functions from the cochlear compound action potential **(A)**, the inferior colliculus **(B)** and the auditory cortex **(C)** in the awake animal in response to 1 kHz (left column) and 4 kHz (right column) tonal acoustic stimulation. By recording prior to lesion induction (thick lines), 3 days (thin lines), 2 weeks (circles) and 5 weeks (triangles) after lesioning, there was found to be a maintained suppression of evoked response amplitudes in both the cochlea and the inferior colliculus; paradoxically, IHC ablation yielded a marked enhancement of evoked response functions recorded from the auditory cortex. Modified with permission from Qiu et al. (2000).

Nerve response properties may nevertheless be affected in other ways. By chronically monitoring the CAP over a period of weeks, long term (≤5 weeks post-exposure) reductions in the maximum evoked CAP magnitude can be observed for chinchillas with selective IHC ablation (Qiu et al., 2000) (Figure 3). Since the CAP represents the summed activity of multiple fibers, reduction in the steepness (or gain) of the nerve's rate-level function (RLF) may ostensibly derive from either, or each, of two sources: a similar reduction in gain of the RLFs of individual fibers; or a loss of fibers encoding high intensity sounds (Liberman, 1982; Taberner and Liberman, 2005). Each is likely, particularly in conditions of permanent threshold elevation and the associated cochlear pathophysiology. Certainly, acoustic exposure, yielding a range of threshold elevations, was reported to mediate a reduction in the gain of individual fiber responses to a spectrally diverse range of stimuli, regardless of the fiber's baseline spontaneous firing rate (SFR) (Heinz and Young, 2004; Heinz et al., 2005).

By contrast, even if cochlear trauma is minimal, such as following recovery from an acoustically-induced “temporary” hearing loss, a frequency-selective deactivation of ANFs responsive up to high intensities may occur, causing a shift in the distribution of ensemble spontaneous activity toward higher firing rates (>20 sp/s) (Furman et al., 2013) (Figure 1). This pattern of deafferentation has been associated with a depression in IHC ribbon synapse density (see above), and has been observed to arise in presbyacusis (Schmiedt et al., 1996). Thus, cochlear trauma is potentially linked with a degradation of high intensity coding beginning at the level of the auditory nerve, in spite of possible threshold preservation. As a matter of comparison, although the ototoxic and threshold-elevating aspects of salicylate administration are well-documented (Cazals, 2000), its effects on spontaneous rate are ambiguous. Auditory nerve hyperactivity has been reported following salicylate infusion (e.g., Ruel et al., 2008) or systemic treatment (Evans and Borerwe, 1982; Cazals et al., 1998). From the available data, however, this phenomenon may simply be a by-product of extremely high systemic concentrations, far beyond those generally observed in human patients (Stolzberg et al., 2012).

### Ventral and dorsal cochlear nucleus

As a major site of ANF afferent termination, neurons in the VCN would be expected to display profound changes in activity, putatively compensating for the proposed reduction in peripheral drive. Given the high SFRs of ANFs, even the “silent” loss of high-threshold (low spontaneous rate) fibers might have profound effects on the baseline activity of VCN cells. SFRs of cells in the VCN appear to be fundamentally determined by ANF activity (Koerber et al., 1966). Although it is marked by a frequency-specific increase in threshold to tonal stimulation and apparent loss of evoked sensitivity in mouse presbyacusis (Willott et al., 1991), the VCN nevertheless may display an increase in contralaterally driven activity (Bledsoe et al., 2009).

The idea that the VCN expresses post-lesion response enhancement through plastic remodeling is intriguing. In the case of a partial cochlear lesion achieved mechanically or acoustically, a significant elevation of spontaneous discharge developed (Vogler et al., 2011). Interestingly, only in the former case was this increase limited cochleotopically, and in terms of the cell subpopulations concerned, with primary-like and onset response-type neurons displaying the largest enhancements (Vogler et al., 2011). This seems to confirm the reported enhancement of tone-evoked activity by transient acoustic trauma in those neural types (Boettcher and Salvi, 1993). By comparison, when identified units of the VCN were recorded following permanent hearing loss, primary-like neurons in particular displayed diminished rate-level function gain (RLF), whereas non-primary chopper type cells consistently exhibited monotonic steep RLFs (Cai et al., 2009). This remodeling of neuronal input/output functions is potentially a consequence of diminished inhibitory function, in conjunction with adaptive hyperactivity after diminished afferent drive. If this is the case, the VCN may be a major station for providing an augmented feedforward representation of cochlear activity to higher levels of the auditory pathway (Noreña, 2011; Noreña and Farley, 2013).

As the other major subdivision of the CN, and thus in receipt of ANF afferent terminals, neurons in the dorsal cochlear nucleus (DCN) have been extensively investigated for changes in excitability under a variety of traumatic conditions. A significant SFR increase in DCN cells develops within a few (2–5) days following cochlear trauma, and persists for weeks thereafter (Kaltenbach and McCaslin, 1996; Zhang and Kaltenbach, 1998; Kaltenbach and Afman, 2000; Kaltenbach et al., 2000; Chang et al., 2002; Finlayson and Kaltenbach, 2009; but see Ma and Young, 2006). The intrinsic excitability of fusiform cells (the major excitatory output from the DCN) is elevated bilaterally even after unilateral noise exposure (Brozoski et al., 2002). This activity change mirrors those seen in the response profiles of fusiform cells (Caspary et al., 2005) and glycinergic cartwheel cells (Caspary et al., 2006) during presbyacusis onset in rats. Although amplified responses may be derived from systemic inhibitory dysfunction (Middleton et al., 2011), the existence of a correlation between OHC disruption and DCN spontaneous discharge indicates that the onset of hyperactivity within the DCN is probably the result of a confluence of factors (Kaltenbach et al., 2002; Rachel et al., 2002).

### Inferior colliculus

At the level of the inferior colliculus (IC) in the auditory midbrain, the predictability of trauma-related neuronal excitability modulations becomes far less concrete, with outcomes varying as a function of exposure mechanism and recovery time. Spontaneous firing rate (SFR) elevations have been characterized in the days-to-weeks following an acoustic trauma (Dong et al., 2009; Mulders and Robertson, 2009; Manzoor et al., 2013; Robertson et al., 2013), complete cochlear deafferentation (Moore et al., 1997) and acutely after high-dose salicylate injection (Jastreboff and Sasaki, 1986; but see Ma et al., 2006). Generally, SFR develops in a manner that is correlated with those cochlear regions displaying equivalent bandpassed CAP threshold increases, with IHC and/or OHC loss (Willott et al., 1988a; Mulders et al., 2011). The origin/s of this hyperexcitability remains under debate, however. First, the peripheral origin of spontaneous rate enhancement appears to be contingent on some degree of OHC ablation only. This is suggested by carboplatin injection in chinchillas—selectively lesioning the IHC population—which had no effect on SFR of IC neurons 7–9 months after trauma. By contrast, such elevations did emerge following acoustic or cisplatin trauma, each of which produces some degree of OHC damage (Bauer et al., 2008).

Moreover, sectioning of the cochlear nerve has been reported to normalize trauma-related hyperactivity in the IC central nucleus (CNIC) (Mulders and Robertson, 2009), as has lesion of the DCN (Manzoor et al., 2012). These data suggest that the IC may chiefly adopt the evolved, persistent hyperactivity of the DCN (Zacharek et al., 2002; Zhang et al., 2006). Nevertheless, longer term observations have revealed that the IC may transition through a period of SFR lability prior to a more permanent acquisition of hyperactivity. By contrast with ipsilateral cochlear nerve lesions performed at 2–6 weeks after acoustic trauma, when identical auditory neurectomy was repeated at 8–12 weeks, IC hyperactivity remained (Mulders and Robertson, 2011). Evidently, then, the IC represents another auditory centre that displays compensatory upregulation of resting activity in response to trauma (Robertson et al., 2013).

Available data remain controversial regarding the evoked input/output gain function changes in IC neurons. In the extreme case of complete cochlear ablation, little-to-no effect upon the response properties of the contralateral CNIC during acoustic stimulation of the intact ear was initially reported (Nordeen et al., 1983). Strong response enhancement has, however, more recently been demonstrated, when the impacts of similar cochlear lesion models were investigated acutely and after three months (Popelár et al., 1994; McAlpine et al., 1997). In addition to an enhancement of the CNIC neural responses to ipsilateral acoustic stimulation, an increase in evoked activity on the contralateral side develops in the next three months after the cochlear insult. The dynamic range of recorded units were also found to be selectively broadened. This adaptive reweighing of collicular responses to favor input from the remaining ear is thought to be motivated by contralateral deafferentation removing the afferent drive to which these cells are predominantly responsive.

How, then, do the collicular cells respond to acoustic stimulation following an incomplete lesion? While response thresholds may be transiently elevated, largely as a function of OHC damage or apoptosis (Salvi et al., 1990), in the case of OHC preservation, threshold shifts are undetectable (McFadden et al., 1998; Alkhatib et al., 2006). When suprathreshold stimulation levels are considered, whereas maximum response amplitude enhancement has been documented (Willott and Lu, 1982; Salvi et al., 1990; McFadden et al., 1998), more often the loss of IHC integrity appears to engender a reduction in neuronal suprathreshold responsivity (McFadden et al., 1998; Qiu et al., 2000; El-Badry and McFadden, 2007) (Figure 3). This is akin to the effects of cochlear administration of salicylate (Sun et al., 2009a), which is thought to be active against inhibitory pharmacology (Su et al., 2009). In addition, there is a reduction in the proportion of non-monotonic-type rate level functions (Alkhatib et al., 2006), a response pattern that is characteristically reliant upon normal inhibitory activity. In presbyacusis-affected C57Bl/6 mice, similar effects were dependent on cochlear threshold elevation (Willott et al., 1988b), since rate-level function steepening did not emerge in aged CBA/J mice that displayed less marked cochlear degeneration (Willott et al., 1988a). This correlation between peripheral disruption and alterations of rate-level function statistics, plus the preponderance of depressed suprathreshold evoked potential amplitudes, seems to support the conclusion that loss of peripheral normality may selectively depress inhibitory function within the IC.

### Auditory thalamus

The auditory thalamus (medial geniculate body, MGB) is a major lynchpin in the transmission of sensory information to the auditory cortex, and a key mediator of auditory receptive field properties of cortical neurons. Nevertheless the effects of trauma upon MGB neurons have been little explored thus far, particularly in terms of categorical shifts in response properties following auditory insult.

Suprathreshold neural response latencies are predominantly retained across a range of characteristic tuning frequencies following partial cochlear ablation (Kamke et al., 2003). Yet, indications of excitability shifts were provided by evaluating shifts in the strength of synchronous activation, specifically in the ventral division of the MGB (Sun et al., 2009b). These latter recordings were only reported for a single animal at each of 4 and 24 h after trauma. Nevertheless, they are congruent with the only comprehensive evaluation of insult-related response plasticity in the auditory thalamus (Richardson et al., 2012, 2013). By performing patch recordings made in the MGB of aged rats, substantial reductions in the amplitude of the evoked phasic and tonic GABAergic current density throughout the MGB were seen. Moreover, region-specific modulations of spontaneous inhibitory activity appeared to develop during the aging process. This was seen in comparisons of the dorsal (non-lemniscal) division of the MGB, which underwent significant reductions in spontaneous inhibitory postsynaptic current, and the ventral (lemniscal) MGB, where a paradoxical enhancement developed in parallel (Richardson et al., 2013). On the basis of these limited but intriguing observations, it appears that the MGB, as a whole, displays the characteristic perturbation of GABAergic inhibitory signaling seen in other auditory centers.

### Auditory cortex

By the level of primary auditory cortex (A1), basal activity and suprathreshold responses might each be conceivably elevated, based on its afferent inputs. Certainly, aging has been demonstrated to bring about spontaneous rate increases in cortical units in a number of species, including rat (Hughes et al., 2010) and rhesus macaque (Juarez-Salinas et al., 2010). A similar effect appears to manifest after acoustic overexposure (Noreña and Eggermont, 2003, 2006), though at a delay of a few hours after trauma, leading to sustained augmentation across A1 for weeks to months (Seki and Eggermont, 2003; Engineer et al., 2011).

In the case of acoustic trauma, cross-correlation analysis revealed coincident enhancement of neural spiking synchrony. This elevation in synchrony was particularly prevalent in those regions that displayed some amount of threshold elevation post-traumatically (Noreña and Eggermont, 2006). Based on the observation that this aspect of neural activity was enhanced almost instantaneously following trauma (Noreña and Eggermont, 2003), and prior to the onset of spontaneous rate enhancements, correlated network spiking may be acting as a precursor to the ongoing plastic modulation of single cell and network response properties.

In each of the studies to have evaluated the effects of salicylate administration on auditory cortex basal activity, the route of administration appears to affect the outcome, even if salicylate may qualitatively raise thresholds, like certain other forms of trauma. Depression of neural activity ensues following direct application of salicylate to the cortical surface (Lu et al., 2011). Systemic administration has, by contrast, been associated with wholesale depressions in spontaneous rate (Yang et al., 2007; Zhang et al., 2011) or simultaneous elevations and depressions in spontaneous discharge (Ochi and Eggermont, 1996; Lu et al., 2011). This latter phenomenon appears to indicate a process of normalizing the mean ensemble discharge by augmenting the spontaneous rate of minimally active units, and depressing the basal discharge of those units with high spontaneous activity. These shifts in spontaneous rate develop in the absence of short-term modulation of intracortical spike synchrony (Ochi and Eggermont, 1996). This is certainly intriguing, given that synchrony is, apparently, enhanced almost immediately after (non-salicylate-based) cochlear trauma.

The idiosyncratic nature of these perturbations, relative to other experimentally induced insults, is further evidence that salicylate is likely to have complex, multifactorial effects throughout the auditory system, both peripheral and central. For example, enhanced driven neural activity is consistently observed across the tonotopic extent of the cortical surface even when salicylate is administered by different routes, such as systemically (Qiu et al., 2000; Yang et al., 2007; Sun et al., 2009a; Deng et al., 2010; Noreña et al., 2010; Lu et al., 2011; Zhang et al., 2011) (Figure 3), or directly to the cortical surface (Lu et al., 2011)—the latter approach being unlikely to bring about changes within the cochlea itself. The likelihood that this stably inducible effect is mediated via a suppression of GABAergic signaling efficacy is strongly suggested by the absence of any such enhancement when agents that positively modulate GABA pharmacology were coadministered (Sun et al., 2009a; Lu et al., 2011).

If, on the other hand, cortical evoked activity patterns are contrasted with those in the cochlear nuclei or the inferior colliculi induced by peripheral pathology, A1 appears to display changes that are uniquely indicative of early adaptation to deprived or modulated sensory input. In one case, recordings from cortical neurons were collected during the 50 h following cochlear ablation (Moore et al., 1997). In that experiment, there was a gradual decline in response thresholds to best-frequency stimulation of the ipsilateral (unlesioned) ear, suggesting adaptive reweighing to favor conserved inputs was occurring.

The evolution of this reweighing appears to vary according to the cortical region affected most severely by deafferentation, leading to cases in which driven activity may simultaneously increase and decrease within the same animal (Noreña et al., 2010). Still, a greater proportion of monotonic rate-level functions are seen in neurons tuned to frequencies outside of the region of cortex responding to the lesioned cochlea (Noreña and Eggermont, 2003) (this region of the auditory lemniscal pathway has been dubbed the “lesion projection zone,” or LPZ Schmid et al., 1996; Calford, 2002). This observation, alongside that of enhanced evoked (Engineer et al., 2011) or maximum firing rates, may well be illustrative of enhanced excitatory activity as well as reduced inhibitory regulation (e.g., Rajan, 2001).

**Figure 4:**
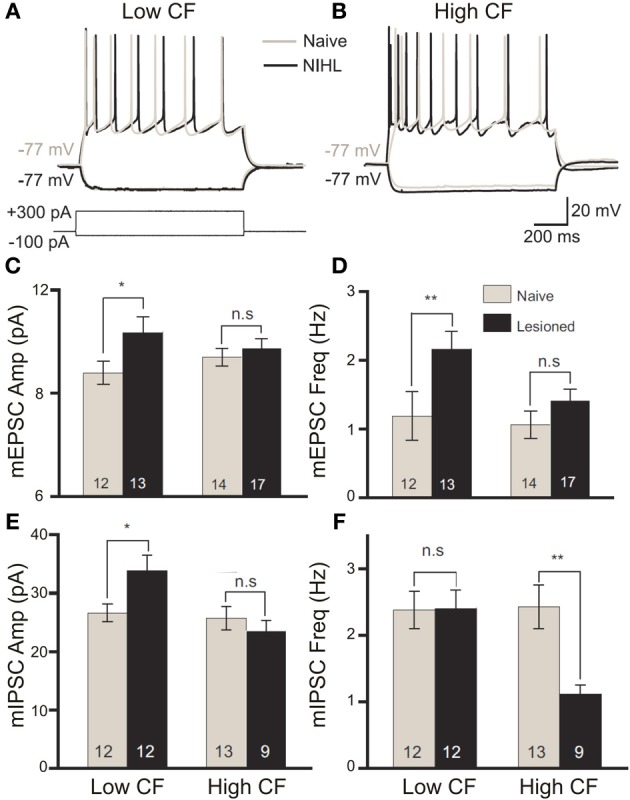
**The balance of excitation and inhibition is differentially modified by band-limited acoustic trauma according to the frequency tuning of cortical neurons**. Following acoustic trauma (123 dB SPL, 4 kHz, 7 h) of adult rats, *in vitro* patch recordings from primary auditory cortex layer II/III pyramidal neurons were performed to evaluate relative changes in excitability between neurons with low- or high-categorized characteristic frequency tuning. In response to square current pulse injection, low-CF neurons showed no enhancement in the number of spikes fired **(A)**, whereas high-CF cells displayed significantly elevated excitability **(B)**. Modified with permission from Yang et al. (2012). On recording miniature excitatory postsynaptic potentials (mEPSCs), there was a significant increase in the amplitude **(C)** and frequency **(D)** of mEPSCs in low-CF neurons only following trauma. Similar analysis of miniature inhibitory postsynaptic potentials (mIPSCs) revealed a significant trauma-driven elevation of mIPSC amplitude in low-CF neurons **(E)**, while the frequency of mIPSCs was significantly depressed in high-CF cells **(F)**. Modified with permission from Yang et al. (2011). ^*^p ≤ 0.05; ^**^p ≤ 0.01.

Evidence that the mechanistic underpinnings of this putatively homeostatic reweighing may operate for both excitatory and inhibitory synaptic signaling has been suggested in single cell recordings from animals with noise-induced threshold elevations. Whereas cells outside of the LPZ displayed proportional enhancement of excitatory and inhibitory activity, those neurons that underwent peripheral deafferentation instead displayed reweighing toward more excitatory responses (Yang et al., 2011) (Figure 4). This effect was correlated with reduced tonic GABA_A_ signaling and GAD65 expression in the LPZ. Reduced inhibitory signaling efficacy is common to a rodent presbyacusis model (Llano et al., 2012), as is the description of significantly depressed tonic GABA_A_ activity at the level of the ventral MGB (Richardson et al., 2013). As such, the elevation of response magnitudes in the auditory cortex, rather than simply being the result of passive unmasking of pre-existing excitatory inputs, may be the result of homeostatic reweighing of physiological excitatory/inhibitory balance toward network hyperexcitability. In future work, it will be important to disentangle the degree to which this homeostatic plasticity is endogenous to each auditory centre recorded from in a trauma model, or if the apparent excitability changes are simply inherited from elsewhere. It is likely that a combination of each is the case, though recent data nevertheless indicate the existence of categorical differences between centers with respect to neuronal excitability (e.g., Cai et al., 2014).

## Insult-mediated changes in neural spectrotemporal receptive fields and networks

Insults to the auditory system are capable of disrupting or even abrogating the network response features to stimuli that vary in spectral and temporal content. Efforts at characterizing these network-level modulations have typically taken two approaches: defining the spectral bandwidth characteristics of auditory cells in the tonotopically organized centers of the lemniscal auditory pathway, and their temporal tuning characteristics in response to more complex, time-varying stimuli, such as amplitude- or frequency-modulated sounds.

### Frequency receptive field remapping

**Figure 5:**
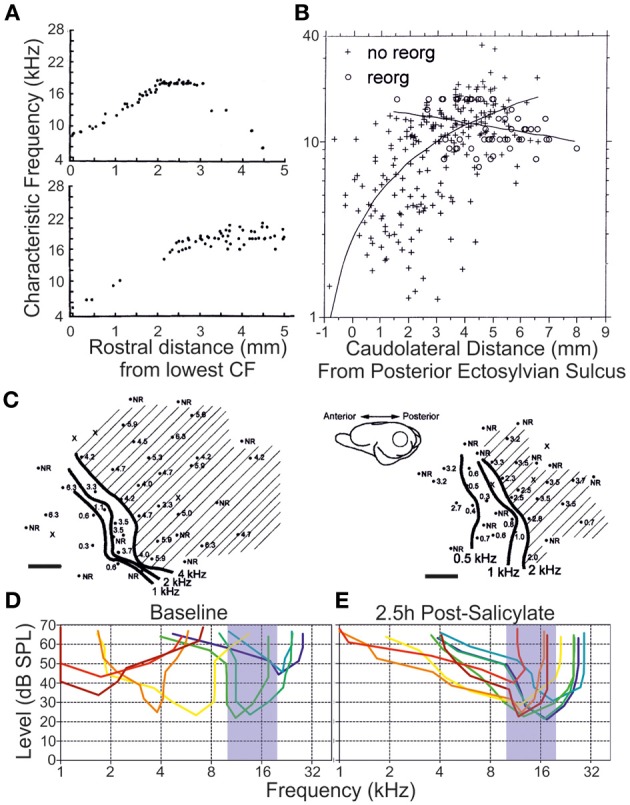
**Remapping of the tonotopy in the primary auditory cortex following a variety of peripheral insults. (A)** A flattening of the linear progression of characteristic frequency (CF) tuning when units were recorded along the dorsoventral axis of primary auditory cortex is observed following partial mechanical lesion of the cochlea in adult cats (individual subjects, upper and lower. Each datapoint represents a single unit CF. Modified with permission from Rajan et al. (1993). **(B)** A similar flattening of tonotopic progression is demonstrated as a function of distance along the cortical surface dorsoventrally in adult cats exposed to narrowband-passed acoustic trauma (crosses), compared with naive controls (circles). Modified with permission from Seki and Eggermont (2002). **(C)** In individual adult chinchillas (left, right) exposed to amikacin-induced basal cochlear hair cell lesions, there is a rearrangement of cortical responses to preference low characteristic frequency responses at regions of the cortex normally responsive at threshold to higher frequency stimuli (shaded region). Scale bars 1 mm. Modified with permission from Kakigi et al. (2000). **(D)** When individual multiunits were isolated in the adult rat primary auditory cortex (each unit is a different color), together spanning the hearing range of the animal, **(E)** systemic injection of salicylate (250 mg/kg intraperitoneal) produced a dynamic retuning of multiunits toward a CF range of 10–20 kHz by 2.5 h after injection. Modified with permission from Stolzberg et al. (2011).

Among the earlier explorations of ensemble responses to deafferentation and trauma are the systematic characterizations conducted of the tonotopic remapping that develops following the insult. This phenomenon (reviewed in Calford, 2002; Irvine, 2007; Kilgard, 2012) concerns the representation of the cochlear frequency domain along an axis of a lemniscal auditory centre that is modified to produce an enhanced sensitivity to one or more frequency bands—effectively to the exclusion of others. Pioneering work from Irvine and colleagues in the guinea pig (Robertson and Irvine, 1989), later extended to the adult cat (Rajan et al., 1993), unveiled a systematic flattening of the primary auditory cortex tonotopic map that was contralateral to partial cochlear damage (Figure 5). This damage was characterized by complete hair cell destruction with limited (<5%) retention of spiral ganglion afferents. In these experiments, a notable change in neural frequency receptive field properties emerged upon comparing acoustic stimulation to the ipsilateral and contralateral ears: whereas in the naive animal the monaural tonotopic maps were effectively in register for each form of tonal stimulation (within ±0.03 kHz), an asymmetry of responsiveness following peripheral lesion developed such that only the contralateral frequency response map was flattened.

Similar, high-frequency, chemical spiral ganglion lesions may also induce tonotopic remapping process, being refined over the course of months to yield enhanced cortical representation of low frequency sounds (Schwaber et al., 1993; Kakigi et al., 2000) (Figure 5). Notably, however, the phenomenon may be observed within days to weeks following selective deafferentation (Seki and Eggermont, 2002; Noreña et al., 2003; Engineer et al., 2011; Yang et al., 2011). Frequency reweighing in the cortex following salicylate has also been described (Stolzberg et al., 2011). Rather than displaying shifts toward the low-frequency edge of the retained normal inputs, instead neurons became abnormally sensitive to stimuli between 10 and 20 kHz at physiological thresholds. In these experiments, recorded units displayed substantially broadened bandwidths, implying that a large degree of their sensitivity to pre-exposure CFs was presumably retained (Stolzberg et al., 2011). It is also rather striking that both salicylate exposure (Deng et al., 2010) and acoustic trauma (Yin et al., 2008) each yield an apparently equivalent reduction in the cortical gap-detection threshold for stimulation off-channel, i.e., completely outside of the region displaying peripheral threshold elevation. This might suggest that each insult commonly disrupts a frequency-insensitive mechanism, or mechanisms, critical to the precise encoding of temporally salient information.

In cases of acute remapping like those described above, the loss of feedforward drive may simply intensify the *relative* expression of certain frequencies that had previously occupied peripheral regions of each unit's frequency response area. While evoked response thresholds are elevated, albeit moderately, by the induction of trauma, the overexpression of particular bandwidths tends to occur outside of the LPZ. Elevated driven response magnitudes, in addition, would imply the existence of a short-term plastic unmasking of afferent inputs that affects the relative interaction of excitatory and inhibitory receptive fields (Rajan, 2001). This phenomenon may develop even in the absence of CF shift (Rajan, 1998). In the chronic phase, however, the redevelopment of normalized peak thresholds and sharp tuning, with little variance of frequency receptive field width across the cortical surface, advocates for intrinsic plastic phenomena, adaptively reweighing neural sensitivity in favor of the remaining inputs.

Furthermore, there exists the possibility that cortical remapping may be inherited, in part, from plasticity in subcortical centers. Recordings made in the ventral division of the MGB contralateral to the cochlear lesion indicate the existence of putative tonotopic remapping in that centre (Kamke et al., 2003). The importance of the thalamocortical circuitry in potentially mediating high-level remodeling of receptive fields after peripheral receptor organ damage, as distinct from simple rostral transmission of deafferented, occasionally silenced (Rajan and Irvine, 1998), projections can be hypothesized.

As a point for comparison, the smooth, linear progression of tonotopy along the dorsoventral axis of the CNIC was predictably flattened after a cochlear lesion, producing an expanded region in which the characteristic frequency of collicular cells matched that of the lower edge of the LPZ (Irvine et al., 2003). However, there was a pronounced dichotomy among those neural units measured, in terms of the stimulus intensity required to drive activity. Although many neurons displayed markedly raised thresholds, akin to those observed in the VCN, others presented driven onset activity at ecologically relevant sound levels. While there exists the possibility of selective remodeling of neural connectivity, the fact that similar tuning patterns were observed in the acute phase following high-frequency spiral ganglion lesions (Snyder et al., 2000, 2008; Snyder and Sinex, 2002) might suggest a release from inhibition of previously subthreshold inputs coding for infra-LPZ frequencies. Indeed, these inputs are highly convergent, and partially comprise suprathreshold ipsilateral projections, which in the lesioned animal are enhanced approximately 5-fold (Irvine et al., 2003; Izquierdo et al., 2008).

Interestingly, such adaptive remodeling may not be universal. In recordings from A1 in the short term, tonotopic remapping has been found to normalize over the course of a week post-trauma (Ahlf et al., 2012). Moreover, following a low-frequency permanent deafferentation, ipsi- and contralateral cortical tuning were found, by 6 months post-insult, to be brought back into register (in contrast with the conserved tuning described above) (Cheung et al., 2009). The mechanism by which this occurred involved an elevation of the thresholds and reweighing of the receptive field characteristics of the cortex ipsilateral to the lesion. This is of particular concern for studies into the longer-term perceptual effects of monaurally limited hearing loss, given that this process develops despite the apparent disadvantages involved with accommodating for the lesioned ear bilaterally, and may be important in considering the utility of fitting hearing aids as early as possible in unilaterally impaired audiological patients. It is, also notable that the frequency receptive fields of infragranular cortical neurons were acutely unstable in aged rats (Turner et al., 2005). Concurrent with a remapping of frequency representations at the cortical surface associated with presbyacusis-mediated cochlear threshold shifts (Willott et al., 1993), an age-related change to the distribution of receptive field shapes of infragranular cortical neurons appears to develop, with overrepresentation of “complex,” non-V-shaped fields (Turner et al., 2005). How this may affect frequency-dependent perception remains to be seen.

### Changes in properties of network responses to temporally complex stimuli

In the context of complex stimulus processing, it is possible that more fundamental shifts in neural output may distort the representation or encoding of stimuli in “higher” cortical centers. Recordings in the rhesus monkey have highlighted an age-related loss of hierarchical abstraction across cortical fields, such that in young monkeys the spatial, directional tuning acuity of neuronal responses is amplified from A1 to the more secondary caudolateral field (area CL), whereas such refinement is absent in older animals (Juarez-Salinas et al., 2010). These effects derive from reductions in inhibition to off-target locations in both CL and A1 (yielding broader receptive fields), as well as a reduction in onset latency in CL, suggesting that plasticity may selectively arise in the primate corticothalamic system, thus enhancing the representation of primary afferent stimuli in non-primary cortical fields (Engle and Recanzone, 2012). To the extent that such cortical remodeling may have the capacity for normalization, recent experiments have demonstrated that following auditory-driven behavioral training, aged rats displayed rectification of the tonotopic map found in A1, as well as in neuronal spectrotemporal response properties (De Villers-Sidani et al., 2010). The correlation between these functional improvements in A1 network activity with a post-training enhancement in parvalbumin levels—a marker of fast spiking inhibitory neurons important to perception- and learning-derived network plasticity (e.g., Donato et al., 2013), which undergoes downregulation following age-related hearing loss (Martin del Campo et al., 2012)—is further indicative of the possible maladaptive effects generated by inhibitory dysregulation.

The suggestion that temporal processing deficits may endure at the level of the IC, having also been documented in the cochlear nuclei, is apparent even from ABR analysis, which provides a surrogate for synchronous network activation (Buchwald and Huang, 1975). In long-latency ABR waveforms, which correlate with auditory midbrain activity (Melcher and Kiang, 1996; Melcher et al., 1996), clear age-dependent modulations in network response timing emerged among older animals (Nozawa et al., 1996). Although no such age-related differences were found during frequency-modulated stimulation of collicular cells (Lee et al., 2002), markedly worsened synchrony to modulated stimulation in aged animals was effected by the addition of background noise (Parthasarathy et al., 2010) and by varying the stimulus modulation depth (Parthasarathy and Bartlett, 2011). Regarding modulation rate, substantial deficits are evidently present at higher modulation rates (Parthasarathy and Bartlett, 2012), and indeed for collicular multi-units, gap-detection thresholds were significantly elevated in aged CBA/CaJ mice (Walton et al., 1998).

These temporal processing deficits align conceptually with observations from a two-tone suppression protocol that indicated significant differences in post-stimulus suppression and facilitation among collicular neurons (Finlayson, 2002). Here, some aged cells displayed abnormally long suppression time constants, indicative of altered encoding of temporally precise stimuli. Since a reduction in rise times to sinusoidally amplitude-modulated (SAM) stimuli also developed in aged CBA mice (Simon et al., 2004), inhibitory dysregulation affecting IC information processing may be a factor common to auditory trauma phenotypes.

It is, however, important to note that specific changes appear to develop across the different IC subdivisions. External cortex (ECIC) neurons in F344 rats displayed a proportional shift toward non-monotonic response when presented with contralateral stimuli (Palombi and Caspary, 1996a), although response bandwidths and net dynamic range of ECIC and CNIC cells failed to change over time with aging. This absence of effective age-related response modulation in F344 rats was also reported during binaural stimulation, typically associated with ipsilaterally-derived suppression of contralaterally-derived excitation (Palombi and Caspary, 1996b). However, when the same authors investigated responses to temporally-complex SAM stimuli, both ECIC and CNIC neurons showed clear divergence from those response distributions recorded in young rats (Shaddock Palombi et al., 2001). Such data concur with experiments in the mouse, in which dramatic differences in preferred modulation rate emerged in aged animals, reducing from 200 to 70 Hz in the upper quartile of units recorded (Walton et al., 2002). Despite failing to express consistent tonotopic changes, used elsewhere as a metric for neuroplastic rearrangement, the clear changes to the temporal coding operations of the IC, which may have behavior- and context-specific impacts with age (Harrison, 1981; Brown, 1984), are strong evidence of cellular- and network-level imbalances with functional consequences.

### Altered cross-modal sensitivity of the auditory system following trauma

The occurrence of receptive field expansion in the trauma-exposed auditory system is therefore interesting, since integration across neuronal computational modes may have unexpected network or perceptual consequences. Indeed, aside from displaying compromised acoustic responsivity, the bilaterally deafferented auditory cortex comprised a significant proportion of neurons that exhibited salient responses to somatosensory inputs (Allman et al., 2009). However, the proportion displaying verifiable multisensory integration, under conditions of selectively preserved auditory input, is in fact diminished relative to control animals (Meredith et al., 2012).

**Figure 6:**
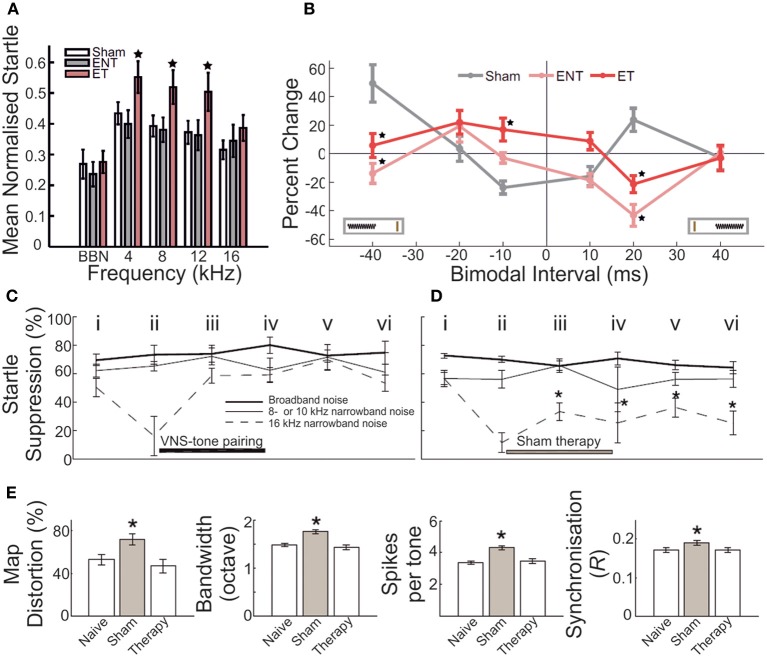
**Gap-prepulse inhibition of the acoustic startle reflex (GPIAS) in rodents acts as a marker for underlying neurobiological changes following auditory insult. (A)** In a cohort of adult guinea pigs, acoustic overexposure (1/4 octave band noise centered at 7 kHz, 93 dB SPL, 2 h; twice-exposed, separated by 6–8 weeks) yielded a subset of animals that displayed significantly decreased normalized startle response inhibition for background masking in the 4–16 kHz bandwidths. Sham controls = white bars; GPIAS-unimpaired animals = gray bars; GPIAS-impaired animals = pink bars. Star = significantly different (*p* < 0.05) from other bars in the same frequency band **(B)** On the basis of categorization according to the presence of GPIAS deficits, this subset of animals displayed significantly different stimulus-timing dependent plasticity functions for bimodal somatosensory-auditory stimulation compared with both sham controls, and exposed but unimpaired animals. Sham = gray; GPIAS-unimpaired = pink; GPIAS-impaired = red. Modified with permission from Koehler and Shore (2013a). **(C–E)** Following exposure to acoustic trauma (octave band noise centered at 16 kHz, 115 dB SPL, 1 h), adult rats underwent daily pairing of tonal stimuli outside the 8–10 kHz frequency bandwidth, either with vagal nerve stimulation (VNS) **(C)** or sham stimulation **(D)**. VNS-tone pairing was found to produce rapid and long-lasting remediation of GPIAS impairments for 8–10 kHz background masking; sham-tone pairing produced no such improvement, with startle suppression ratios remaining depressed throughout the testing period following trauma. Time points: i, before trauma; ii, 4 weeks after exposure; iii, 10 days after initiation of VNS-tone or sham-tone pairing therapy; iv, 20 days after therapy initiation; v, 1 week after conclusion of therapy; vi, 3 weeks after conclusion of therapy. Broadband noise (thick line), non-tinnitus frequency (thin line), putative tinnitus frequency (dashed line). **(E)** In addition, VNS-tone pairing was found to remediate and normalize with respect to control measurements, aspects of primary auditory cortex neuronal operation which had been significantly enhanced following trauma, including tonotopic map distortion, frequency receptive field bandwidth, evoked spike response number, and neural spike-timing synchrony. For each bar plot, leftmost bar = naive; middle bar (gray) = sham-tone pairing therapy; rightmost bar = VNS-tone pairing therapy. Asterisk = significantly different (*p* < 0.05) compared with controls. Modified with permission from Engineer et al. (2011).

In related work from Shore and colleagues, an enhancement of somatosensory input into the DCN driven by traumatic threshold elevations has been described (Shore et al., 2008). This modified sensory afferent input in the DCN is accompanied by alterations of the typical stimulus timing-dependent plasticity rules for bimodal integration during successive auditory/somatosensory stimulation (Dehmel et al., 2012b; Koehler and Shore, 2013a,b) (Figure 6), generally producing a long-term enhancement of unimodal acoustically evoked activity. Given that earlier descriptions of the effects of cochlear trauma concluded the absence of plastic remapping in the DCN (Kaltenbach et al., 1992; Rajan and Irvine, 1998), it is worthwhile, in future work, to consider alternative plasticity mechanisms, including cross-modal reorganization, which may develop post-traumatically throughout the auditory neuraxis.

## The functional and behavioral implications of trauma-driven auditory plasticity

A constellation of neural changes evolves in parallel throughout the auditory system in response to the challenge posed by the introduction of mechanically-, pharmacologically-, acoustically-, or aging-derived insults (Table 1). These changes can be classed as dynamic modulations of spontaneous and driven neural activity, the underpinnings of which appear to rely upon putatively homeostatic gain-modulation mechanisms that affect the extent of neural excitability, and selectively modulate the balance of excitation and inhibition. Changes at the single neuron and network processing levels can be demonstrated *in vivo* (e.g., Noreña, 2011; Yang et al., 2011, 2012) and by *in silico* modeling (Dominguez et al., 2006; Schaette and Kempter, 2006, 2008, 2012; Chrostowski et al., 2011; Tass and Popovych, 2012; Schaette, 2013). Although these neurophysiological sequelae are becoming better understood, it remains an ongoing problem as to whether the various components of central modulation, at different levels of the auditory pathway, might translate into perceptual abnormalities, like tinnitus, that could interrogated by behavioral testing.

A major point of inquiry in current auditory neuroscience thus seeks to explain how it is that (subjective) tinnitus, being the perception of an auditory stimulus in the absence of an environmental equivalent, may come about in the wake of auditory trauma of a variety of forms, and manifest in only a proportion of any tested cohort (e.g., Eggermont and Roberts, 2004; Roberts et al., 2010; Noreña and Farley, 2013). In addition, concerns have recently been raised regarding the capacity to behaviorally test for the presence/absence of some abnormal positive percept. A possible division might be drawn between tinnitus as distinct from other correlated perceptual abnormalities, such as hyperacusis (Baguley, 2003), or from the predicted outcomes of hearing loss in the absence of any positively generative perceptual changes (Eggermont, 2013).

To that extent, a number of questions ought to be addressed: what classes of behavioral changes have been conclusively shown to develop following the introduction of some specific auditory insult (in the absence of *a priori* assumptions regarding the perceptual correlates of these behavioral data)? What are the neurobiological changes that have been shown to develop *in parallel* with these behavioral changes? And is it possible to conclude some effective *functional* link between certain aspect/s of the underlying insult-affected neurobiology and a categorical class of phantom percept?

### Cochlear lesion-induced behavioral threshold modulations

Of the behavioral studies that have been conducted related to sensorineural auditory insults in adult mammals, the range of observed effects correlates well with predictions made according to early physiological studies. In particular, those studies aimed at disambiguating the changes that develop in the periphery and at the level of the auditory nerve have been informative. Some insults have little effect on detection thresholds—for example, partial section of the auditory nerve in cats was found to have no impact on intensity thresholds except in the most severe of cases (Neff, 1947; Schuknecht and Woellner, 1953, 1955). Clearly, in the absence of complete destruction of afferent cochlear transmission, a small population of surviving neurons is sufficient to provide a basis for accurate and sensitive detection profiles. Conversely, damage at the cochlea is associated with behavioral threshold elevation in a manner related primarily to the extent of OHC loss (Ryan and Dallos, 1975; Hawkins et al., 1976; Ryan et al., 1979). After that kind of trauma, auditory nerve fiber tuning curves displaying marked loss of both sharp peak tuning and sensitivity by approximately 40 dB SPL at maximum.

In lieu of OHC loss, there is relatively good preservation of audiometric thresholds (Lobarinas et al., 2013b), whereas frequency discrimination is apparently reliant on effective preservation of the IHC population (Nienhuys and Clark, 1978). It is likely that the majority of peripherally-mediated insults affect one or both subtypes of hair cell population, even in cases in which the animal's electrophysiologically characterized thresholds are normalized following trauma (Kujawa and Liberman, 2009). As such, it might be predicted that the perceptual experience of an adult mammal subject would be affected in some fashion.

More recently, the focus of behavioral characterization has included the evaluation of changes in temporal processing ability, primarily (though not exclusively) in the form of testing gap-detection thresholds following insult. Electrophysiological data have demonstrated that neurometric gap detection thresholds deteriorate in the awake animal with aging (e.g., Recanzone et al., 2011) or acoustic trauma (e.g., Yin et al., 2008) (c.f. unilateral cochlear ablation, in which acoustic stimulation of the intact ear produced normal neurometric functions Kirby and Middlebrooks, 2010). Such neural changes appear to be behaviorally reflected by reductions in psychometric discrimination performance using gap-in-noise (Giraudi-Perry et al., 1982; Salvi and Arehole, 1985; Rybalko and Syka, 2005; Gold et al., 2013) or amplitude-modulated stimuli (Henderson et al., 1984). Performance of these operant behavioral paradigms is certainly reliant upon the normal function of the central auditory system for effective fine-scale perceptual discrimination of temporally-modulated stimuli. This can be reasoned from the effects of severe bilateral decortication/auditory cortical lesioning, which do not abolish task performance, but nonetheless significantly raises the detection thresholds of adult animals with otherwise normal hearing function (Ison et al., 1991; Kelly et al., 1996; Threlkeld et al., 2008). On the basis of these ablation/inactivation studies, it is not unreasonable to conclude that the accurate perception of the temporal fine structure of the stimuli, and so the performance of operant tasks reliant upon that perception, is expected to be affected following sensorineural insults that perturb normal auditory functionality.

As an additional measure of temporal processing abnormalities, a number of studies have leveraged various paradigms concerning the prepulse inhibition (PPI) of the acoustic startle response in small mammals. The behavior is reflexive, and thus can be recorded in the absence of prior training. It is notable, however, that there are indications of species-related efficacy, or rather lack thereof, of the response (Gruner, 1989; Pilz and Leaton, 1999). Nevertheless, it can be optimized for investigating aspects of an animal's perceptual experience. In particular, suppression of the acoustic startle using a gap-in-noise prepulse (gap-mediated prepulse inhibition of the acoustic startle, or GPIAS) can be modulated according to the specific experimental conditions imposed. These include the temporal interval between the prepulse and the startle stimulus, the amplitude of the startle stimulus, and the spectral content of the background noise masker (Longenecker and Galazyuk, 2012; Lobarinas et al., 2013a; Hickox and Liberman, 2014).

The introduction of an insult akin to those described that affected operant conditioning-derived deficits of gap detection may be interrogated by way of the acoustic startle response. From recordings performed either in the same subjects, or subjects exposed to identical stimuli which impaired GPIAS, aspects of subcortical temporal processing plasticity were found to be selectively affected in animals with gap-detection impairments, as distinct from subjects which have undergone comparable insult exposure, or naive controls (Koehler and Shore, 2013a) (see Figures 6A,B). Also, recordings in the auditory cortex have suggested that GPIAS may be a useful metric for evaluating insult-mediated changes in cortical activity. In particular, there was correlation between levels of neural synchrony and frequency tuning, and remediation of behavioral deficits observed to occur following a paired vagal nerve stimulation protocol (Engineer et al., 2011) (see Figures 6C,D).

Recent data have suggested that the susceptibility of subjects to impaired gap-mediated startle suppression following acoustic trauma might be related to baseline levels of excitability of the auditory neuraxis. Differential outcomes were found among subjects with variable levels of resting cortical firing rates and evoked activity, manifesting in addition as instability of cortical tonotopy (Ahlf et al., 2012). This variable expression of neural excitability is almost certainly related to the plasticity phenotype of animals in various insult conditions. Indeed, systemic salicylate injection, which is understood to affect neural excitability (see above), has been recognized as effective in similarly modulating the suppression efficacy, largely in parallel with upregulation of neural gain in the auditory cortex (Deng et al., 2010; Sun et al., 2014).

Given the extensive excitatory/inhibitory remodeling that is associated with aging (Caspary et al., 2008), it is possibly unsurprising that GPIAS impairment has also been demonstrated in a mouse model of presbyacusis. In this model, behavioral dysfunction was correlated with cortical inhibitory dysfunction demonstrated using novel functional imaging techniques (Llano et al., 2012). GPIAS behavioral impairment is undoubtedly a useful indicator of certain aspects of the possible underlying pathophysiology that affects neuroplastic changes after trauma. It may not be premature to suggest that the GPIAS represents an effective metric of auditory temporal processing deficits that occur subcortically. Indeed, cortical deficits may also modulate subcortical processing of the reflex circuitry by way of strong corticofugal projections (Bajo and King, 2012), which may also undergo insult-driven plastic remodeling. The role of these projections in the context of insult-mediated plasticity is largely unknown, and requires exploration in future experiments.

### Perceptual phantoms and proposed behavioral correlates

The development of methods for reliably demonstrating a tinnitus-like percept in experimental animals represents another major research topic still in progress. The existence of tinnitus is, by convention, categorically different from the perception of silence. It is thus not unreasonable that a popular approach has sought to detect the phantom percept using operant conditioning paradigms to test perception. Often—but not always—under negative motivation, these require the animal to choose between a background noise stimulus, and either the absence of an acoustic stimulus, or the presence of some acoustic stimulus whose spectral content is deemed similar to the tinnitus percept (Jastreboff et al., 1988; Bauer and Brozoski, 2001; Heffner and Harrington, 2002; Guitton et al., 2003; Rüttiger et al., 2003; Yang et al., 2007, 2011; Sederholm and Swedberg, 2013).

Intuitively, experimental approaches of this kind are likely to be successful in revealing a subjective abnormality in the animal's perceptual state. The evaluation of the decision criterion, which is contingent upon the subject's integration of stimulus- and network-driven auditory activity, is likely to take into account the possible existence of a phantom percept. Indeed, the various demonstrations that long-known tinnitus inducers in human patients, such as high-dose salicylate (Cazals, 2000), bring about behavioral disruptions under such operant conditioning paradigms, indicate that the pursuit of phantom percepts in animals may not be wholly intractable.

More recently, the GPIAS paradigm has been proffered as a potential tool for behavioral interrogation of the phantom percept. Under the working framework that the presence of the tinnitus will internally mask, or “fill-in,” the presence of the gap in noise acting to suppress the startle response, a cohort of animals with diminished startle suppression as a function of some auditory insult might be revealed (Turner et al., 2006, 2012). The paradigm has definitely yielded interesting and promising data regarding the behavioral correlates of neurophysiological modulation—related to changes in tuning, evoked and spontaneous single unit activity, and broader network correlational aspects of auditory function—and has been validated in relation to other reported tinnitus detectors (Bauer and Brozoski, 2001; Yang et al., 2007). However, the likelihood that the technique acts as a reliable indicator of tinnitus proper remains under debate (Eggermont, 2013).

If indeed tinnitus “fills in the gap,” it is puzzling how it is that modifying the prepulse-startle inter-stimulus interval can renormalize GPIAS activity in animals whose suppression behaviors are selectively compromised (Hickox and Liberman, 2014). In those cases, IHC ribbon synapse pathology was comparable with the pathology displayed by subjects with operant-demonstrated behavioral deficits (Rüttiger et al., 2013; Singer et al., 2013). Moreover, gap-mediated startle suppression was absent during salicylate overdose when the gap-in-noise contained slowly ramped offset windows (Sun et al., 2014). This is despite the same animals displaying effective startle suppression when the gap on/offset ramp characteristics are otherwise modified (Sun et al., 2014). Observations of this kind may indicate that tinnitus, if present, fails to fill in the silent period consistently in the manner suggested in other reports of tinnitus-like behavior.

**Figure 7:**
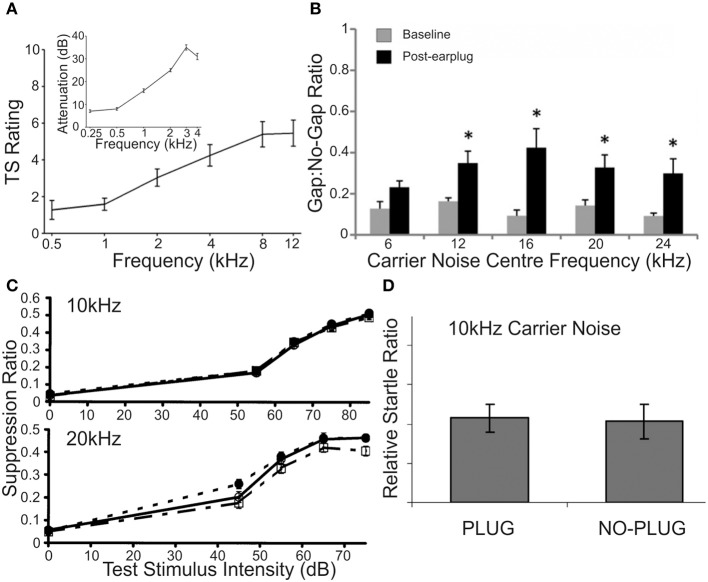
**Unilateral conductive hearing loss yields conflicting reports of tinnitus generation in adult human and non-human normal-hearing subjects. (A)** Chronic (>7 days) insertion of an earplug into one ear produced subjective reports of tinnitus in 10/18 human listeners, evaluated on a tinnitus similarity likeness scale; the group average tinnitus likeness indicated the development of a low-pass sensation peaking between 8 and 12 kHz. Inset is the attenuation function for earplug insertion, indicating a sloping attenuation of high-frequency stimuli up to ~30 dB SPL for stimuli >3–4 kHz. Modified with permission from Schaette et al. (2012). **(B)** Using GPIAS as a surrogate reporter of tinnitus presence in adult normal-hearing rats, unilateral earplug insertion also revealed tinnitus-like symptoms, as a reduction in startle-suppression efficacy for gaps inserted into carrier noise (60 dB SPL) narrow-bandpassed at 12, 16, 20, and 24 kHz. No such reduction developed for a 6 kHz narrowband carrier. Gray bars = baseline startle suppression ratio; black bars = plug startle suppression ratio; asterisk = significant difference within frequency band compared to baseline (*p* < 0.05). Modified with permission from Lobarinas et al. (2013a). **(C)** Conversely, testing of the effects of unilateral plugging on an operant sound detection task for stimuli presented at 10 kHz (upper) and lower (20 kHz) in adult rats showed there to be no effect upon detection functions for either stimulus bandwidth; by contrast, animals that underwent acoustic overexposure (narrowband noise centered at 16 kHz, 105 dB SPL, 1 h) displayed marked reduction in detection functions at 20 kHz, interpreted as the presence of tinnitus whose spectral content matched that frequency band. Control = open circles; normal-hearing with plug = filled squares; acoustic trauma without plug = open squares. Modified with permission from Bauer and Brozoski (2001). **(D)** A similar evaluation of the effects of unilateral earplug in adult rats found no difference between GPIAS functions in the same subjects prior to and following earplug insertion. Background noise was centered at 10 kHz, at which plug insertion produced a mean attenuation of 22 dB SPL. Modified with permission from Turner et al. (2006).

A further worry is the apparent susceptibility of GPIAS to modulation by unilateral earplug insertion (Lobarinas et al., 2013a) (Figure 7). This result has been interpreted as a false-positive detection of tinnitus in non-tinnitus animals, on the grounds that transient earplugging failed to reveal a tinnitus-like behavior under operant conditioning detection (Bauer and Brozoski, 2001). According to recent human data, chronic (>7 days) unilateral earplug insertion can induce positive phantom percepts in a majority of young, healthy listeners (Schaette et al., 2012). Animal behavioral studies have thus far only investigated transient earplugging effects, and so are not wholly comparable to the chronic earplugging condition of Schaette and colleagues. Yet, it is notable that enhanced neural synchrony has been postulated as a potential correlate of tinnitus in the auditory cortex (Noreña et al., 2003; Eggermont and Roberts, 2004; Roberts et al., 2010), developing more or less instantaneously following sensorineural auditory trauma (but not, it seems, following salicylate Ochi and Eggermont, 1996). If the phantom perceptual outcome of plugging operates by way of a similar functional framework as is to blame in “sensorineural” tinnitus (Schaette and Kempter, 2006; Schaette et al., 2012), it is not unlikely that transient tinnitus-like percepts may be inducible in a subset of subjects with a unilateral earplug. This may (Lobarinas et al., 2013a) or may not (Bauer and Brozoski, 2001; Turner et al., 2006) manifest during “tinnitus-sensitive” behavioral testing.

The emergence of non-specific gap-detection deficits has recently been demonstrated among a subset of tinnitus patients (Fournier and Hébert, 2013; but see Campolo et al., 2013), and thus such perceptual abnormalities may well form part of the tinnitus syndrome in animal models thus far reported. It is, however, insufficient to rely on deficits of this kind to categorically define the presence of tinnitus, even in the context of physiological and anatomical features that have been related to the disease in the past. What is required is the development of behavioral paradigms that more specifically disentangle the presence of a phantom percept from the spatiotemporal processing deficits that may arise from auditory trauma, neuroplastic changes, or otherwise. Almost certainly, the implementation of these paradigms would rely upon baseline measurements being obtained in the same animal population as is subsequently exposed to trauma, enabling repeated-measures statistical tests to be leveraged. In addition, the exploration of novel behavioral and pathological models will expand our capacity to interrogate the perceptual effects of central auditory changes which develop following trauma. Prior to causally relating behavioral phenotypes to particular neurophysiological modulations to that have been labeled “tinnitus-like,” it would be constructive to develop a fuller appreciation of the neurobiological changes that consistently develop in the wake of tinnitus-related effectors, irrespective of the proposed instantiation of the percept itself.

### The future of insult-related plasticity and perceptual changes

Recent circumspection has called into question the proposed relationship between certain neurophysiological changes and the development of auditory phantoms (Eggermont, 2013). Contrasting changes to spontaneous discharge rates were found following different insults that each yielded equivalent behavioral results (e.g., Bauer et al., 2008). There is a lack of correspondence between remediation of behavioral changes and spontaneous rate elevations, despite other neurophysiological features undergoing normalization (Engineer et al., 2011), and an apparently paradoxical depression or amplification of spontaneous rate in certain studies of salicylate administration (Eggermont and Kenmochi, 1998; Yang et al., 2007; Zhang et al., 2011). Similarly, the use of tonotopic remapping as a tinnitus-specific metric can be problematic due to the contrast between short term reversals in tonotopic map plasticity (Ahlf et al., 2012) compared with therapy-related tonotopy restoration (Engineer et al., 2011) in animals with “tinnitus-like” behavior. Indeed, in human patients, both the presence (Mühlnickel et al., 1998; Weisz et al., 2005) and the absence (Langers et al., 2012) of tonotopic remapping have been reported, further frustrating the interpretation of animal data.

While historically less focused upon in research than tinnitus, hyperacusis is gaining traction as a potential sequela of the trauma-induced maladaptive plasticity syndrome. Recent reports have linked the state of enhanced auditory sensitivity to the development of tinnitus in human patients (Dauman and Bouscau-Faure, 2005; Fournier and Hébert, 2013; Hébert et al., 2013). Behavioral data have suggested that the possibility for detection of a hyperacusis-like state in animal models may exist via the emergence of enhanced gain of the acoustic startle reflex, and its attendant prepulse suppression. This type of behavior has been noted following acoustic trauma (Dehmel et al., 2012a; Sun et al., 2012; Chen et al., 2013; Pace and Zhang, 2013; Hickox and Liberman, 2014), salicylate treatment (Turner and Parrish, 2008; Sun et al., 2009a), or aging (Ison et al., 2007).

It is possible that the neurobiological factors mediating the maintenance of hyperacusis are distinct from, though likely related to, those inducing tinnitus. Computational (Zeng, 2013) and molecular models (Knipper et al., 2013) each have sought to assign specific network modulation patterns, particularly those associated with aberrant central signaling gain changes, to the occurrence of post-traumatic hyperacusis. Certainly, evidence of enhanced driven neural activity has been noted subcortically (Cai et al., 2009) and cortically (Qiu et al., 2000; Yang et al., 2007; Sun et al., 2009a, 2012) following various forms of auditory overexposure (see above). It is intriguing to ask how it is that the auditory system's innate gain control systems may be perturbed to potentially generate an abnormal perceptual experience. Indeed, the observed disparities between cortical and subcortical gain functions may in part be explained by hierarchically distinct, physiological differences in gain control (Rabinowitz et al., 2011, 2012). As lines of inquiry, these certainly warrant further examination in integrated behavioral and neurophysiological models, chiefly to answer key concerns regarding the development of the condition, observed with (Dauman and Bouscau-Faure, 2005) and without (Gu et al., 2010) peripheral threshold elevations.

Fruitful investigation is likely to be achieved by evaluating the causes underlying changes to neural gain and network spiking synchrony following some tinnitus-related insult, particularly with respect to rebalancing of excitatory/inhibitory signaling mechanisms (Knipper et al., 2013). A broader consideration of multiregional network effects throughout the auditory pathway is required. It is necessary to highlight the temporally-defined differential effects of post-insult regional disruption, particularly at the level of the auditory brainstem and midbrain (Brozoski and Bauer, 2005; Mulders and Robertson, 2009, 2011; Brozoski et al., 2012). In addition, the necessary involvement of ascending and descending projections throughout the auditory neuraxis in mediating physiological and behavioral changes following auditory insult remain underexplored (Bajo et al., 2010; Bajo and King, 2012). In the absence of any effective and reliable therapeutic measures being available for tinnitus treatment in human sufferers (Baguley et al., 2013; Langguth et al., 2013), it is clear that the insult-related plastic processes related to, and distinct from, perceptual phantom generation must be unraveled for clinical progress to proceed.

### Conflict of interest statement

The authors declare that the research was conducted in the absence of any commercial or financial relationships that could be construed as a potential conflict of interest.
